# One-year follow-up of the CAPSID randomized trial for high-dose convalescent plasma in severe COVID-19 patients

**DOI:** 10.1172/JCI163657

**Published:** 2022-12-15

**Authors:** Sixten Körper, Beate Grüner, Daniel Zickler, Thomas Wiesmann, Patrick Wuchter, Rainer Blasczyk, Kai Zacharowski, Peter Spieth, Torsten Tonn, Peter Rosenberger, Gregor Paul, Jan Pilch, Joachim Schwäble, Tamam Bakchoul, Thomas Thiele, Julian Knörlein, Matthias M. Dollinger, Jörg Krebs, Martin Bentz, Victor M. Corman, Dzenan Kilalic, Gerlinde Schmidtke-Schrezenmeier, Philipp M. Lepper, Lucas Ernst, Hinnerk Wulf, Alexandra Ulrich, Manfred Weiss, Jan Matthias Kruse, Thomas Burkhardt, Rebecca Müller, Harald Klüter, Michael Schmidt, Bernd Jahrsdörfer, Ramin Lotfi, Markus Rojewski, Thomas Appl, Benjamin Mayer, Philipp Schnecko, Erhard Seifried, Hubert Schrezenmeier

**Affiliations:** 1 Institute for Clinical Transfusion Medicine and Immunogenetics Ulm, German Red Cross Blood Transfusion Service Baden-Württemberg-Hessen and University Hospital Ulm and Institute of Transfusion Medicine, University of Ulm, Ulm, Germany.; 2 Division of Infectious Diseases, University Hospital and Medical Center Ulm, Ulm, Germany.; 3 Department of Nephrology and Medical Intensive Care, Charité - Universitätsmedizin Berlin, corporate member of Free University Berlin, Humboldt-Universität zu Berlin, and Berlin Institute of Health, Berlin, Germany.; 4 Department of Anesthesiology and Intensive Care Medicine, Phillips-University Marburg, Marburg, Germany.; 5 Institute of Transfusion Medicine and Immunology, German Red Cross Blood Transfusion Service Baden-Württemberg-Hessen, Medical Faculty Mannheim, Heidelberg University, Germany.; 6 Institute of Transfusion Medicine and Transplant Engineering, Hannover Medical School, Hannover, Germany.; 7 Department of Anesthesiology, Intensive Care Medicine and Pain Therapy, University Hospital Frankfurt, Goethe-University, Germany.; 8 Department of Anesthesiology and Critical Care Medicine, Carl Gustav Carus University Hospital, Technische Universität Dresden, Dresden, Germany.; 9 Transfusion Medicine, Medical Faculty Carl Gustav Carus, Technische Universität Dresden and German Red Cross Blood Donation Service North-East gGmbH, Dresden, Germany.; 10 Department of Anesthesiology and Intensive Care Medicine, University Hospital Tübingen, Tübingen, Germany.; 11 Department of Gastroenterology, Hepatology, Pneumology and Infectious Diseases, Klinikum Stuttgart, Stuttgart, Germany.; 12 Institute of Clinical Hemostaseology and Transfusion Medicine, Saarland University Hospital, Homburg/Saar, Germany.; 13 Institute of Transfusion Medicine and Immunohematology, German Red Cross Blood Transfusion Service Baden-Württemberg – Hessen, Frankfurt, Germany.; 14 Institute of Clinical and Experimental Transfusion Medicine, University Hospital Tübingen, Tübingen, Germany.; 15 Institute of Transfusion Medicine, University Hospital Greifswald, Greifswald, Germany.; 16 Clinic of Anesthesiology and Intensive Care Medicine, University Medical Center of Freiburg, Freiburg, Germany.; 17 Medical Clinic I, Klinikum Landshut, Landshut, Germany.; 18 Clinic for Anesthesiology and Surgical Intensive Care Medicine, University of Mannheim, Mannheim, Germany.; 19 Department of Internal Medicine III, Hospital of Karlsruhe, Karlsruhe, Germany.; 20 Institute of Virology, Charité - University Medicine Berlin, corporate member of Free University Berlin, Humboldt-Universität zu Berlin, and Berlin Institute of Health and German Centre for Infection Research, Berlin, Germany.; 21 Clinic of Internal Medicine II, Ulm University, Ulm, Germany.; 22 Department of Internal Medicine V – Pneumology, Allergology, Intensive Care Medicine, Saarland University Hospital, Homburg, Germany.; 23 Department of Anesthesiology and Intensive Care Medicine, University Hospital Ulm, Ulm University, Ulm, Germany.; 24 Institute of Epidemiology and Medical Biometry, Ulm University, Ulm, Germany.; 25 Alcedis GmbH, Gießen, Germany.

**Keywords:** COVID-19, Therapeutics, Immunotherapy

## Abstract

**BACKGROUND:**

Results of many randomized trials on COVID-19 convalescent plasma (CCP) have been reported, but information on long-term outcome after CCP treatment is limited. The objectives of this extended observation of the randomized CAPSID trial are to assess long-term outcome and disease burden in patients initially treated with or without CCP.

**METHODS:**

Of 105 randomized patients, 50 participated in the extended observation. Quality of life (QoL) was assessed by questionnaires and a structured interview. CCP donors (*n* = 113) with asymptomatic to moderate COVID-19 were included as a reference group.

**RESULTS:**

The median follow-up of patients was 396 days, and the estimated 1-year survival was 78.7% in the CCP group and 60.2% in the control (*P* = 0.08). The subgroup treated with a higher cumulative amount of neutralizing antibodies showed a better 1-year survival compared with the control group (91.5% versus 60.2%, *P* = 0.01). Medical events and QoL assessments showed a consistent trend for better results in the CCP group without reaching statistical significance. There was no difference in the increase in neutralizing antibodies after vaccination between the CCP and control groups.

**CONCLUSION:**

The trial demonstrated a trend toward better outcome in the CCP group without reaching statistical significance. A predefined subgroup analysis showed a significantly better outcome (long-term survival, time to discharge from ICU, and time to hospital discharge) among those who received a higher amount of neutralizing antibodies compared with the control group. A substantial long-term disease burden remains after severe COVID-19.

**Trial registration:**

EudraCT 2020-001310-38 and ClinicalTrials.gov NCT04433910.

**Funding:**

Bundesministerium für Gesundheit (German Federal Ministry of Health).

## Introduction

The use of COVID-19 convalescent plasma (CCP) from patients recovered from a SARS-CoV-2 infection was evaluated in many randomized trials during the pandemic (1–21). The trials were heterogeneous in design and differed in terms of patient populations. Some included patients early in the disease course with mild to moderate disease in an outpatient setting (10, 17–19) and others included hospitalized patients with more severe disease (1–9, 11–16). Some of the trials considered different kinds of risk factors like age or concomitant disease (10). Some nonrandomized trials suggested efficacy in immunocompromised patients (22–25). Of note, the studies differed substantially in quality and quantity of CCP in terms of neutralizing antibody titers and CCP volume and timing of administration (1–19). Patients with severe disease typically had a longer interval since diagnosis. In most of the trials, the primary endpoint was not met and the results were inconclusive. Careful analysis revealed that there is some efficacy of CCP with high titers of neutralizing antibodies, especially when used early in the course of the disease (10, 18, 19). Most trials report outcome data up to 30 days after randomization (2–19). So far, none of them has reported long-term results. Because COVID-19 can lead to long-lasting symptoms, sometimes with significant impairment, termed “long COVID-19” (26–30), it is of great interest to determine whether CCP has any impact on the disease burden in the long term. Immunization by vaccines or infection are effective in the prevention of severe disease. However, so far there is limited information on the vaccination response after the use of CCP.

Here we report the long-term outcome of the CAPSID randomized clinical trial, which included hospitalized patients with severe COVID-19 (1). Hospitalized patients were stratified according to their need for extracorporeal membrane oxygenation, mechanical ventilation, or ICU treatment and then randomized to receive either standard of care or standard of care plus 3 units of CCP on days 1, 3, and 5. The trial showed a trend toward a better outcome in the CCP group but did not reach statistical significance and therefore missed the primary endpoint, which was defined as survival and no longer severe COVID-19 on day +21 after enrollment. In a prespecified subgroup analysis, CCP showed significantly better overall survival (OS) and improvement in other important clinical outcomes among patients who received a larger amount of neutralizing antibodies (1). The per-protocol follow-up time of this first part of the trial was 60 days (median follow-up 60 days) (1). Here, we report a long-term follow-up of the patients (median follow-up 396 days) and also included the CCP donors as a reference group. All CCP donors had experienced only mild to moderate symptoms of COVID-19 prior to CCP donation. To our knowledge this is the first long-term follow-up study of a randomized clinical trial of CCP-treated patients.

## Results

### Study population.

**Figure 1:**
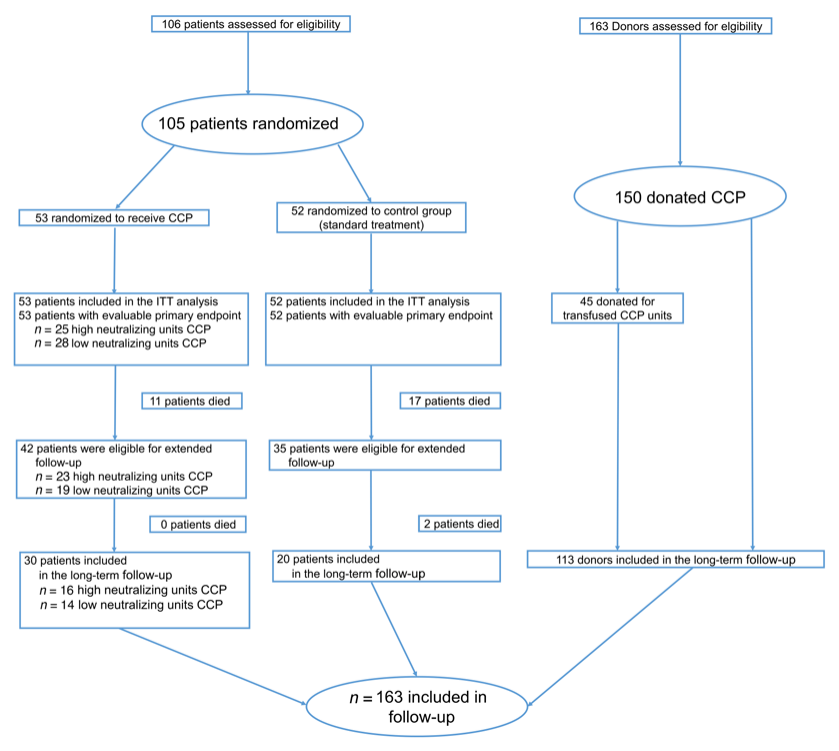
Patient and donor enrollment in the CAPSID trial and the extended follow-up.

One hundred and sixty-three participants were included in the long-term follow-up. Of the 77 survivors (day 60) treated within the CAPSID trial, 50 patients (control group, *n* = 20; high-titer CCP, *n* = 16; low-titer CCP, *n* = 14) (Figure 1) and 113 donors participated in the long-term follow-up evaluation. The median follow-up time for patients was 396 (IQR, 379–417) days after randomization and 519 (IQR, 480–553) days after the first plasmapheresis for donors. Among the included donor population, the median time from symptom onset to first plasmapheresis was 101 days (interquartile range [IQR], 73–124). Among the patient cohort of the extended follow-up, the median time from onset of symptoms to randomization was 8 days (IQR, 5–11). The donors were mostly infected during the first wave in Germany, while the patients were predominantly infected in the second wave.

Baseline characteristics are summarized in Table 1. The donor population was significantly (*P* < 0.0001) younger (42.0 [IQR, 31.0–52.0] years) than the patient population (58.5 [IQR, 54.0–65.0] years). The patient cohort included more males (74%) than the donor cohort (52%). Donors had a significantly lower BMI (25.9 [IQR, 23.3–30.0] kg/m^2^) than patients (29.8 [IQR, 26.6–33.0] kg/m^2^) (*P* = 0.0003). In the donor cohort, mild disease (88.5%) predominated. Of the patients, 68% were graded 5 or higher on the 8-point WHO severity scale (World Health Organization. COVID-19 Therapeutic Trial Synopsis. https://www.who.int/publications/i/item/covid-19-therapeutic-trial-synopsis Updated February 18, 2020. Accessed August 31, 2021.) and 90% reported comorbidities (Table 1).

### Primary and secondary outcomes.

**Figure 2:**
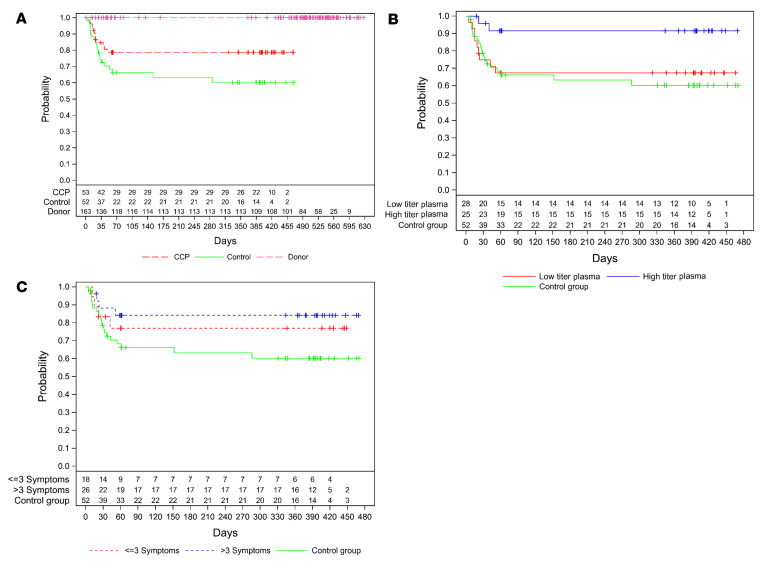
Overall survival. Kaplan-Meier cumulative estimates of probability of overall survival are shown. In all panels, a “+” indicates a censored patient. (**A**) Overall survival of donors (dotted magenta line), control (solid green line), and CCP group (dotted red line). *P* = 0.083 (log-rank test) for CCP versus control group. (**B**) Overall survival compared in the CCP subgroup that received a low cumulative amount of neutralizing antibodies (solid red line), the CCP subgroup that received a high cumulative amount of neutralizing antibodies (solid blue line), and the control group (solid green line). *P* = 0.011 for high amount versus control and *P* = 0.032 for high amount versus low amount (log-rank test). (**C**) Overall survival by amount of donor symptoms in control group (solid green line) patients transfused with CCP from donors with ≤3 symptoms (dotted red line) or transfused from CCP donors with >3 symptoms (dotted blue line). *P* = 0.061 for CCP donors with >3 symptoms versus control (log-rank test).

**Figure 3:**
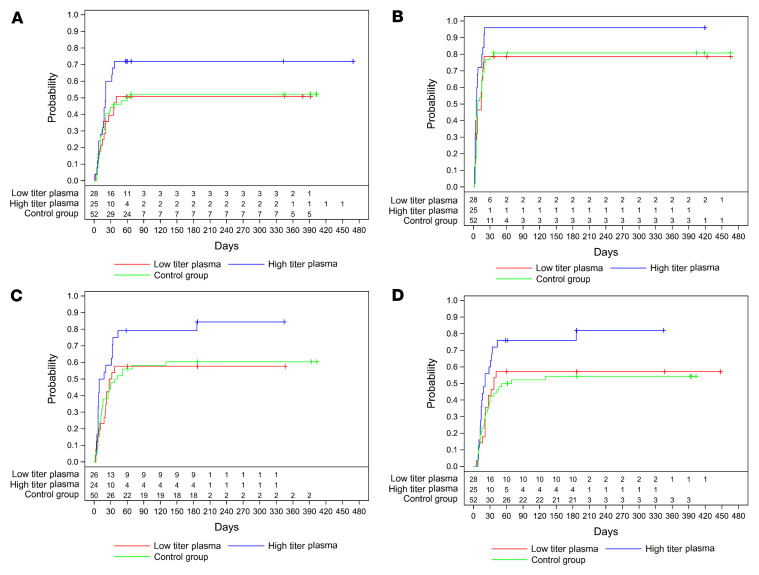
Long-term occurrence of secondary outcomes by amount of transfused neutralizing units. Kaplan-Meier cumulative estimates of probability are shown. In all panels, a “+” indicates a censored patient. (**A**) The key secondary outcome time to clinical improvement compared in the CCP subgroup that received a low cumulative amount of neutralizing units (red), the CCP subgroup that received a high cumulative amount of neutralizing units (blue), and the control group (green line). *P* = 0.088 (log-rank test; high amount vs. control group). (**B**) Time to first negative PCR compared in the CCP subgroup that received a low cumulative amount of neutralizing units (red), the CCP subgroup that received a high cumulative amount of neutralizing units (blue), and the control group (green line). *P* = 0.019 (log-rank test, high amount vs. control group). (**C**) Probability of discharge from ICU compared in the CCP subgroup that received a low cumulative amount of neutralizing units (red), the CCP subgroup that received a high cumulative amount of neutralizing units (blue), and the control group (green line). *P* = 0.025 (log-rank test, high amount group vs. control group). (**D**) Probability of discharge from hospital compared in the CCP subgroup that received a low cumulative amount of neutralizing units (red), the CCP subgroup that received a high cumulative amount of neutralizing units (blue), and the control group (green line). *P* = 0.017 (log-rank test, high amount vs. control group).

No deaths have been reported in the donor population. Two patients in the control group died after day 60 (Figure 2A). The 1-year OS was 78.7% (95% confidence interval [CI], 64.7%–87.6%) in the CCP group and 60.2% (95% CI, 44.4%–72.9%) in the control group (*P* = 0.08). Patients who were treated with a higher cumulative amount of neutralizing antibodies showed a significantly better long-term OS when compared with the control group (1-year OS 91.5% (95% CI, 70.0%–97.8%) versus 60.2% (95% CI, 44.4%–72.9%) (*P* = 0.01) or to the subgroup that was treated with a low cumulative amount of neutralizing units (1-year OS 67.4% [95% CI, 46.6%–81.5%], *P* = 0.03) (Figure 2B). As we have previously shown, the amount of neutralizing antibodies in CCP donors increases with the amount of reported symptoms (31). In a pandemic situation with a newly emerging pathogen, validated tests for neutralizing antibodies are usually not immediately available in the very beginning of the pandemic. Therefore, in this period, criteria other than antibody content might be important for donor selection. We therefore analyzed the OS stratified by the number of symptoms reported by donors. In this evaluation, there is a trend toward a better outcome in patients treated with CCP from donors with more than 3 symptoms compared with the control group (*P* = 0.061) (Figure 2C). However, the difference is not significant and much smaller than in the comparison based on the cumulative amount of transfused neutralizing units (Figure 2, B and C). The better outcome of the subgroup that had received a higher cumulative amount of neutralizing units was confirmed in the final data set, including long-term observation. It shows a significantly shorter time to first negative SARS-CoV-2 PCR (*P* = 0.02), a shorter time to discharge from ICU (*P* = 0.02), and a shorter time to discharge from hospital (*P* = 0.02) (log-rank test; Figure 3, A–D). The primary outcome of the study, i.e., survival and no longer fulfilling criteria of severe COVID-19 on day 21, remained nonsignificant. In the final data set, among those who received a high or low cumulative amount of neutralizing units, the primary outcome occurred in 56.0% and in 32.1%, respectively, and in 30.8% in the control group (*P* = 0.046 high titer vs. control).

### Medical events during long-term follow-up.

Patients reported GI symptoms (including abdominal pain, diarrhea, nausea, weight loss), pulmonary symptoms, dyspnea, pain symptoms, confusion, dizziness, hypersomnia, insomnia, conjunctivitis, or alopecia (Table 2). The control group of patients reported numerically less often GI or pain symptoms than the CCP group (*P* = NS). Pulmonary symptoms were reported in 47% of patients in the CCP group and 70% of patients in the control group (*P* = 0.15), and during extended follow-up supplemental oxygen was needed in 10% of patients in the CCP group but in 30% of patients in the control group (*P* = 0.13) (Table 3). During the extended follow-up period, 18% of patients were hospitalized and 18% of patients needed supplemental oxygen. Twenty percent of patients in the CCP group and 15% of the control group were hospitalized (*P* = 0.724). The duration of hospitalization in the CCP group was 5 (IQR, 3–6) days compared with 15 (IQR, 6–27) days in the control group (*P* = 0.09). The proportion of hospitalization did not significantly differ between patients who had received a high cumulative amount of neutralizing units compared to those treated with a low cumulative amount of neutralizing units (6.2% vs. 35.7%, *P* = 0.07). Radiologic imaging of the chest was comparable between all groups (Table 3).

**Figure 4:**
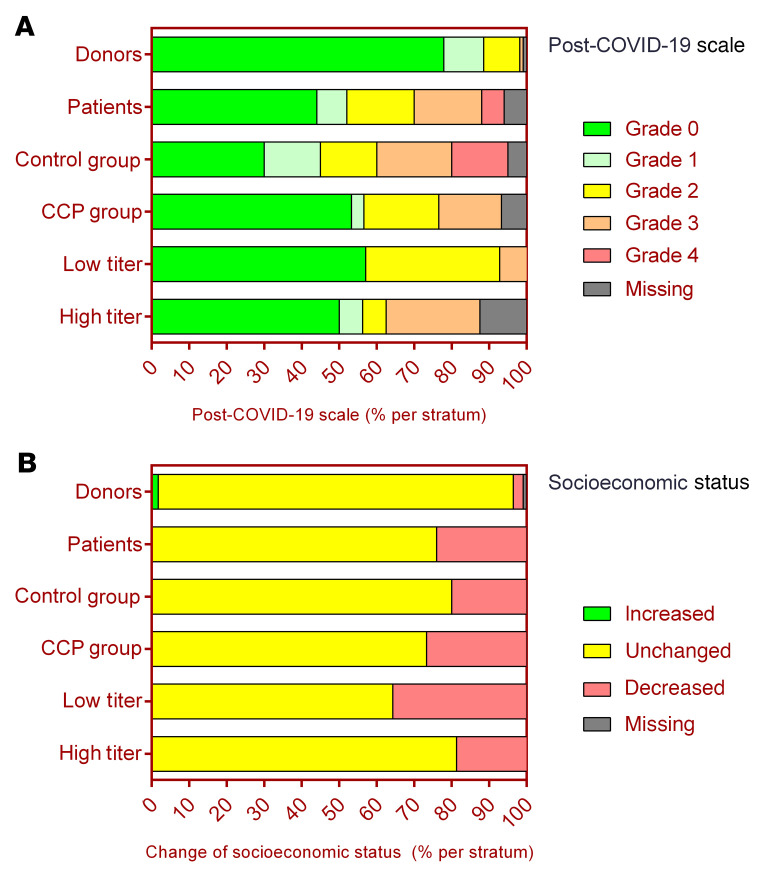
Post–COVID-19 scale and socioeconomic status. (**A**) Relative proportion of donors (upper row), CAPSID trial patients (second row) (*P* < 0.0001 by Fisher´s exact test), patients stratified by randomization group (CCP group and control groups) (middle rows) (*P* = 0.089), and patients who received a high or low amount of neutralizing units (lower rows) (*P* = 0.1304) according to the post–COVID-19 scale from grade 0 to grade 4. (**B**) Relative proportion of donors (upper row), CAPSID trial patients (second row), and patients stratified by randomization group (CCP group and control groups) (middle rows) and patients who received a high or low amount of neutralizing units (lower rows) (*P* = 0.4171) according to their change in socioeconomic status (increased, unchanged, decreased).

Functional limitations assessed by the post–COVID-19 scale (i.e., grade 0 to 4) were reported by 56% of patients (Figure 4A). Grade 2–4 functional limitations were reported by 48% of patients. The number of patients reported to be free of limitations was not significantly different between the CCP group (53%) and control group (30%) (*P* = 0.136) (Figure 4A).

Any medical event during follow-up was reported in 73% of donors and 84% of patients. Events rated as grade 3 or higher occurred in 8% of donors and in 22% of patients (*P* = 0.018). In donors, the most frequent symptoms were neurologic symptoms (57.5%), pulmonary symptoms (37.2%), and pain symptoms (15.9%) (Supplemental Table 1; supplemental material available online with this article; https://doi.org/10.1172/JCI163657DS1). Significantly more patients (18%) than donors (3%) needed oxygen (*P* = 0.0014). Hospitalization for any cause occurred in 7% of donors and in 18% of patients during the extended follow-up period (*P* = 0.051).

The proportion of donors with functional limitations assessed by the post–COVID-19 scale was lower than the proportion in patients (22% vs. 56%, *P* < 0.001), and correspondingly, the subgroup with grade 2–4 limitations was also smaller in donors (10.6% vs. 42%, *P* < 0.001) (Figure 4A).

### Quality of life.

A substantial proportion of patients (24%) reported a decrease in their socioeconomic status during follow-up, with only a slight numerical difference between the CCP group and control group of patients (26.7% vs. 20.0%, *P* = 0.74) (Figure 4B).

**Figure 5:**
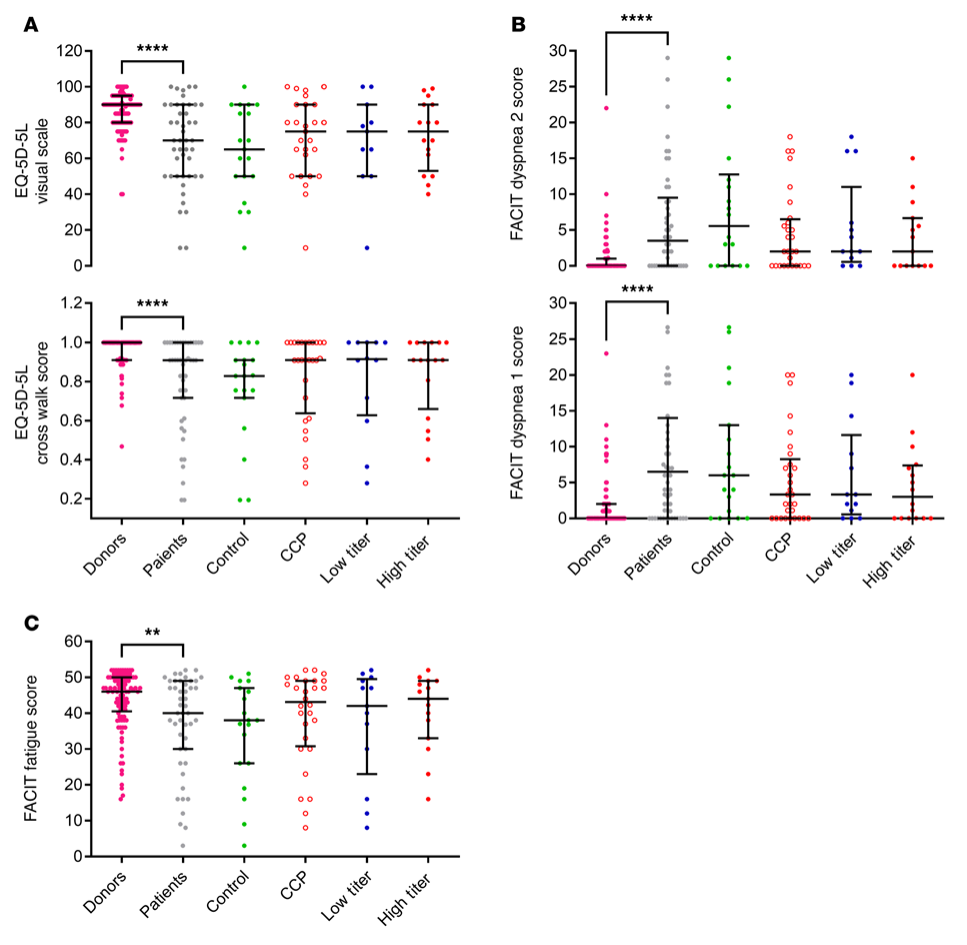
Quality of life score. Data given as median and interquartile ranges. (**A**) EQ-5D-5L visual scale: Donors (*n* = 107) versus patients (*n* = 46) (****P < 0.0001), control group (*n* = 19) versus CCP group (*n* = 27) (*P* = 0.355), and control group versus CCP that received a high cumulative amount of neutralizing units (*n* = 16) (*P* = 0.730). No test was performed for the group that received a low cumulative amount of neutralizing units (*n* = 11). Cross walk score: donors (*n* = 105) versus patients (*n* = 47) (****P < 0.0001), control group (*n* = 19) versus CCP group (*n* = 28) (*P* = 0.280), and control group versus CCP subgroup that received a high cumulative amount of neutralizing units (*n* = 16) (*P* = 0.702). No test was performed for the group that received a low cumulative amount of neutralizing units (*n* = 12). (**B**) FACIT Dyspnea score 1: Donors (*n* = 107) versus patients (*n* = 48) (****P < 0.0001), control group (*n* = 19) versus CCP group (*n* = 29) (*P* = 0.196), and control group versus CCP subgroup that received a high cumulative amount of neutralizing units (*n* = 16) (*P* = 0.518). No test was performed for the group that received a low cumulative amount of neutralizing units (*n* = 13). FACIT Dyspnea score 2: Donors (*n* = 107) versus patients (*n* = 46) (****P < 0.0001), control group (*n* = 18) versus CCP group (*n* = 28) (*P* = 0.15), and control group versus CCP subgroup that received a high cumulative amount of neutralizing units (*n* = 15) (*P* = 0.446). No test was performed for the group that received a low cumulative amount of neutralizing units (*n* = 13). (**C**) FACIT Fatigue score: Donors (*n* = 105) versus patients (*n* = 47) (**P = 0.004), control group (*n* = 19) versus CCP group (*n* = 28) (*P* = 0.306), and control group versus CCP subgroup that received a high cumulative amount of neutralizing units (*n* = 15) (*P* = 0.492). No test was performed for the group that received a low cumulative amount of neutralizing units (*n* = 13). The Mann-Whitney test was used for calculation of *P* values.

Figure 5 shows a summary of total scores of the reported quality of life (QoL) questionnaires. In the EQ-5D-5L questionnaire, the patients of the CCP group reported numerically better outcomes than the control group in all 5 dimensions, i.e., “mobility,” “self care,” “usual activities,” “pain/discomfort,” and “anxiety” (Supplemental Table 2). The dimensions “self-care,” “usual activities,” “pain/discomfort,” “anxiety, and “your health today” were not statistically different between the CCP and control groups, while a significantly higher proportion of patients of the CCP than the control group reported that they have no problems in “walking about” (63% vs. 40%, *P* = 0.0395) (Supplemental Table 3). There was no relevant difference in the EQ-5D-5L items between the patients of the low- and high-titer CCP group (Figure 5A and Supplemental Table 4). The results of the FACIT Dyspnea and FACIT Fatigue questionnaires show similar patterns; scores were numerically better in the CCP group than the control group without reaching statistical significance (Figure 5B and Supplemental Tables 6 and 9). The difference between subgroups by cumulative amount of neutralizing antibodies was small, with a consistent trend for better scores in most of the items in the subgroup that had received a higher cumulative amount of neutralizing units (Figure 5B and Supplemental Tables 7 and 10).

FACIT Fatigue score and the individual items did not differ significantly in the comparisons by randomization group (Supplemental Table 12) and by cumulative amount of transfused units (Supplemental Table 13).

Significantly more patients (24%) than donors (2.7%) reported a decrease in their socioeconomic status during follow-up (*P* < 0.0001) (Figure 4B). In the EQ-5D-5L questionnaire, donors reported significant better outcomes in all 5 dimensions than patients (Supplemental Table 2). The visual scale score of the item “your health today” was significantly higher in donors than in patients (*P* < 0.0001) (Figure 5A and Supplemental Table 2).

In all the QoL questionnaires used in this study, the donors showed significantly better results (Figure 5, A–C).

The score of the FACIT Fatigue scale was significantly higher in donors than patients, indicating less fatigue in the donor group (*P* = 0.0038) (Figure 5C and Supplemental Table 11). The majority of items, in particular “I have energy,” “I am able to do my usual activities,” “I am too tired to eat,” “I need help doing my usual activities,” and “I have to limit my social activity because I am tired” indicate significantly greater impairment in the patient population (Supplemental Table 11).

Because of the differences between the donor and patient population the outcomes might be influenced by other factors than severity of COVID-19. We therefore identified 26 pairs of donors and patients by propensity score matching for the variables age, sex, and BMI (Supplemental Table 16). In this matched cohort, the differences between donors and patients were significant for the change in socioeconomic status and the post–COVID-19 scale (Supplemental Figure 3, A and B), the EQ-5D-5L visual scale and cross walk score (Supplemental Figure 4A), and the FACIT Dyspnea 2 score (Supplemental Figure 4B). FACIT Fatigue and FACIT Dyspnea 1 score did not significantly differ between patients and donors in the propensity score–matched groups (Supplemental Figure 4, B and C).

### Neutralizing antibodies.

**Figure 6:**
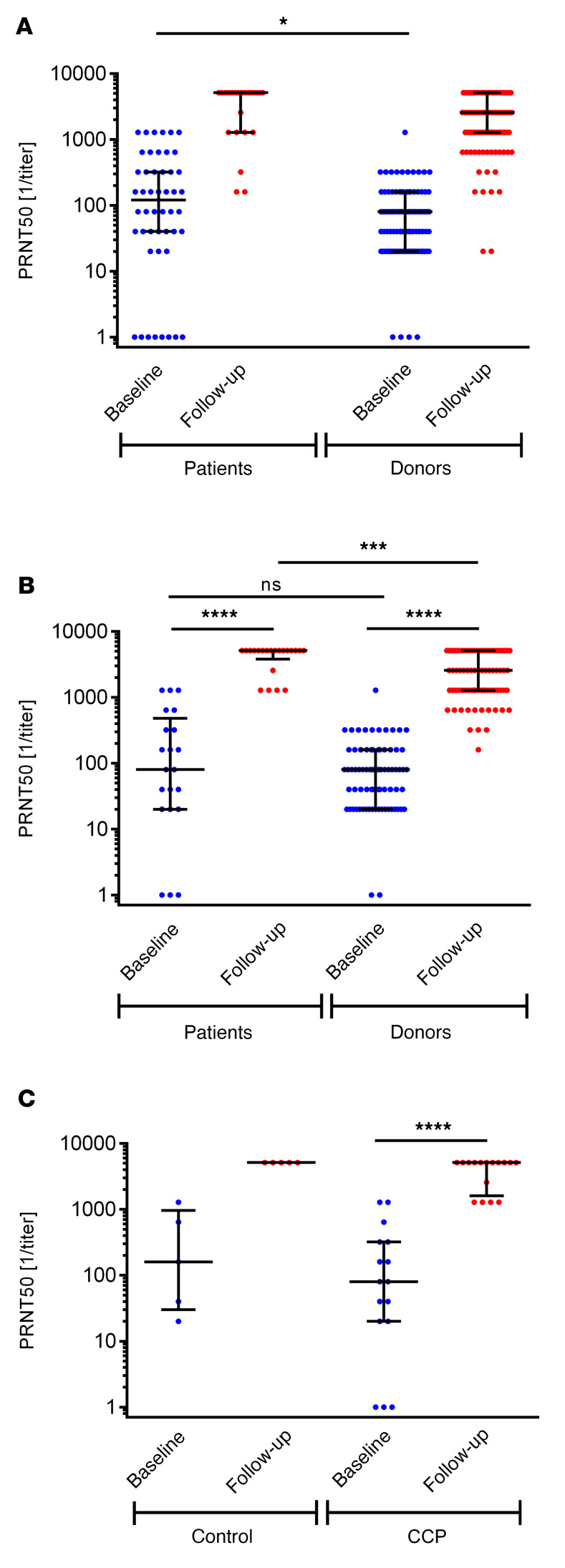
Neutralizing anti–SARS-CoV-2 antibodies (PRNT50) at baseline and last follow-up. (**A**) Neutralizing antibodies of all study participants as available. Follow-up data of patients (*n* = 25) and donors (*n* = 95). Baseline values donors (*n* = 97) versus patients (*n* = 48): *P = 0.045. (**B**) Neutralizing antibodies of vaccinated study participants during follow-up with available baseline and follow-up data. Patients (*n* = 21) baseline versus follow-up values: ****P < 0.0001. Donors (*n* = 76) baseline versus follow up values: ****P < 0.0001. Follow-up values patients versus donors: ***P = 0.0005. (**C**) Vaccinated patients with available baseline and follow-up data by randomization group (CCP [*n* = 16] and control group [*n* = 5]). Baseline versus follow-up in CCP patients: ****P < 0.0001. No test was performed for control because of the low patient number. Horizontal lines indicate the median and interquartile ranges. The Mann-Whitney test was used for calculation of *P* values for unpaired analysis and Wilcoxon’s matched-pair test for comparison of matched pairs.

None of the participants was vaccinated prior to the infection. Most of the patients (86%) and donors (93%) were vaccinated at least once after their infection (Table 1). The median time from infection to first vaccination in patients and donors was 212 (IQR, 189–237) days and 418 (IQR, 390–443) days (*P* < 0.0001). The median intervals from the last vaccination to blood sampling for the follow-up antibody test in patients and donors were 77 (IQR, 15–158) days and 131 (IQR, 31–175) days (*P* = 0.1729). Figure 6 shows the results of the neutralizing titer causing 50% inhibition in the plaque-reduction neutralization test (PRNT50) at baseline or first apheresis and after the long-term follow-up. Among vaccinated participants with available baseline and follow-up data, patient PRNT50 titers increased from 1:80 (IQR, 1:20–1:480) to 1:5120 (IQR, 1:3840–1:5120). A significant increase in PRNT50 titers from 1:80 (IQR, 1:20–1:320) to 1:5120 (IQR, 1:1600–1:5120) was also observed in the patients randomized to CCP (*P* < 0.0001) (Figure 6C).

Anti–SARS-CoV-2 IgG and IgA antibodies measured by ELISA increased significantly after vaccination of patients. The use of CCP seems to have no effect on the increase in IgG or IgA by vaccination (Supplemental Figure 2C).

Baseline PRNT50 titers in patients (1:120 [IQR, 1:40–1:320]) were significantly higher than in donors (1:80 [IQR, 1:20–1:160]) (*P* = 0.045) (Figure 6A). Donor PRNT50 titers increased from 1:80 (IQR, 1:20–1:160) to 1:2500 (IQR, 1:1280–1:5120) (Figure 6A). Vaccinated patients had significantly higher PRNT50 values at follow-up than vaccinated donors (*P* = 0.0005) (Figure 6B).

The baseline anti–SARS-CoV-2 IgG ratio (measured as ratio of optical density by ELISA) of donors (3.8 [IQR, 2.9–5.8]) was comparable to that of patients (3.4 [IQR, 2.2–6.6]) (*P* = 0.5), while the baseline IgA ratio was significantly higher in patients (7.0 [IQR, 2.2–9.0]) compared with donors (2.3 [IQR, 1.3–3.9]) (*P* < 0.0001) (Supplemental Figure 2A). At last follow-up, vaccinated patients and donors had significantly higher IgG and IgA ratios compared with their respective baseline ratios and IgG and IgA ratios did not significantly differ between donors and patients at last follow-up (Supplemental Figure 2B).

### Markers of activation of coagulation and markers of inflammation.

D-dimers as markers of coagulation and C-reactive peptide (CRP), fibrinogen, IL-6, and ferritin as markers of inflammation and pro-NT-BNP remained significantly elevated even more than 1 year after the acute infection in the clinical trial patients (Supplemental Table 14), with no significant difference between the control and CCP groups.

## Discussion

To the best of our knowledge, this is the first randomized clinical trial that reports long-term data on the use of CCP, with a median follow-up of more than 1 year. While many trials of CCP for COVID-19 at different stages of COVID-19 have been published, they report on short observation periods, often just up to about 1 month or less after randomization (2–19). It is evident that during the pandemic, it was important to make the initial results of the trials publicly available as soon as possible. However, the long-term results must also be taken into account, especially as it became clear that long-term complications involving different organ systems after COVID-19 are very common, significantly affect patients’ QoL and also influencing OS (26–30).

The risk of long COVID-19 increases with age, preexisting conditions, and severity of COVID-19 (32–35). Patients who had to be treated in hospital or patients who required intensive care have a higher risk of long COVID-19 than patients with a mild to moderate course who could be treated on an outpatient basis (32, 33). Thus, the risk for the manifestation of long COVID-19 is also increased in the patients in the CAPSID study; the median age in the study was 60 years, all cases had severe COVID-19, and a high proportion of patients (89%) had a previous disease associated with an unfavorable course of COVID-19. Thus, the study population of the CAPSID study represents a group of patients who are particularly at risk for long COVID-19 and who require follow-up for medical reasons.

The lack of knowledge also applies to CCP donors; less is known about the long-term course of former CCP donors. Therefore, we included CCP donors in this analysis to learn more about their long-term disease burden. They also comprised an additional reference group since they had experienced an asymptomatic to moderate COVID-19 as opposed to the CAPSID trial patients who had severe COVID-19. Results of the CAPSID trial based on the initial 2-month observation period and the CCP donor characteristics have been previously published (1, 31). There are several factors that might influence long-term outcome. At the time of the previous analysis, not all patients had reached the respective endpoints (clinical improvement, time to discharge from ICU and hospital). Given the burden of long COVID-19 and persisting organ dysfunction, the outcome might change due to long-term sequela. The enrollment in the CAPSID trial was completed a few days prior to availability of SARS-CoV-2 vaccines in Germany. Also, new variants evolved thereafter. It was not clear how vaccination and potential reinfections would impact the long-term course. Therefore, we considered an extended follow-up necessary. Here, we now provide an update based on a median follow-up of 396 days.

The follow-up demonstrated a long-term OS that was numerically higher in the CCP group compared with the control group, but the difference was not statistically significant. A predefined subgroup analysis of the initial 2-month observation period showed a significant benefit of CCP among patients who received a higher amount of neutralizing antibodies (1). The significant effect of transfusion of a larger amount of neutralizing units tended to be even more pronounced in the long-term observation across several endpoints. In the previous report, the day 60 probability of survival was 91.6% in the subgroup that received a higher cumulative amount of neutralizing antibodies and 68.1% in the control group (*P* = 0.02) (1). Due to additional deaths during extended follow-up, 1-year survival is now 91.5% versus 60.2% (*P* = 0.01) in the high-titer plasma versus the control group. This confirmed the previous report on the importance of the antibody dose (1), in line with other studies that have demonstrated a dose effect (10, 36–38). One study demonstrated that treatment with highly neutralizing plasma was significantly associated with faster virus clearance, but even after adjustment for their pretransfusion endogenous neutralization status, recipients benefitted (38). This observation is in line with the dose effect in the CAPSID trial on several outcomes, including the shorter time to first negative SARS-CoV-2 PCR from a nasopharyngeal swab in the group who received a high cumulative amount of neutralizing antibodies compared with the control group (Figure 3A).

A correlation of the hyperinflammation and cytokine release syndrome with the severity and outcome of COVID-19 has been reported (39–42). Increased levels of several cytokines have been associated with severity (42–49). An antiinflammatory role of CCP independent of its neutralizing antibody content has been demonstrated (50). Neutralizing antibodies as well as reductions in circulating in IL-6 and IFN-γ–induced protein 10 contributed to marked rapid reductions in hypoxia in response to CCP (50).

At the very beginning of the pandemic, reliable quantification of anti–SARS-CoV-2 antibodies was a challenge. We and others have shown some benefit of CCP with high antibody titers, but on the other hand it has been shown that the severity of COVID-19 and the number of symptoms correlates well with the PRNT50 titers in CCP donors. We therefore studied whether the severity of COVID-19, as assessed by the number of symptoms, in the CCP donors correlated with the clinical efficacy of CCP units from those donors. We could show a trend for better outcomes after treatment with CCP from donors with a higher number of symptoms. Based on the lessons learned during the COVID-19 pandemic the selection of high-titer CCP should be based on appropriate antibody assays, if available. However, in the very beginning of a pandemic with a newly evolving pathogen and absence of validated tests for the quantification of the antibodies in CCP, the number of symptoms might provide a surrogate for donor selection in the bridging period until the availability of a validated test. From our data, at least we could not see any harm regarding efficacy or adverse events using such an approach.

It has been demonstrated that the combination of SARS-CoV-2 infection with a SARS-CoV-2 vaccination (in either order) causes both an enhancement of all aspects of the humoral immune response and a broad immune reaction even against new variants (51–55). The underlying mechanisms involve ongoing antibody somatic mutation, memory B cell turnover, and development of antibodies that are resistant to SARS-CoV-2 RBD mutations, including those found in variants of concern (51). Repeated antigen exposure can confer potency, breadth, and resilience to viral escape mutations (56). Therefore, for future CCP programs, priority should be given to superimmunized donors with very high antibody concentration due to previous SARS-CoV-2 infection and vaccination (54, 55, 57).

We used several instruments to assess QoL of donors and patients during the extended observation period (EQ-5D-5L, FACIT Fatigue, FACIT Dyspnea). Notably, the long-term disease burden in the group of donors was not at all negligible, as a substantial subgroup of donors reported slight functional limitations (8.8% to 32.5%) in at least one of the dimensions of the EQ-5D-5L questionnaire, and in all QoL scores there are few donors with results below the median scores of the patients. Fifty-seven percent of donors reported neurologic symptoms, which is comparable to the proportion of the patients reporting neurological symptoms (64%). Conversely, the disease burden in the group of patients was very substantial.

None of the patients improved their socioeconomic status, but a significantly higher proportion of patients than donors reported a socioeconomic status deterioration. A majority of patients reported functional limitations assessed by the post–COVID-19 scale and patients reported consistently more frequently about GI, neurological, and pulmonary symptoms with a higher grade of severity. The CCP group and especially the subgroup that received a higher cumulative amount of neutralizing antibodies showed consistently numerically better results but the differences did not reach statistical significance for the individual item, with the exception of the lower hospitalization rate in the high-dose subgroup. Nevertheless, the trend for fewer constraints in the CCP group was very consistent across 3 different QoL instruments, which cover different dimensions (Supplemental Tables 3, 6, and 9). Also, the proportion of patients without pulmonary symptoms was lower in the CCP group compared with the control group (53% vs. 30%), together with a lower proportion of patients with need for any type of ventilation support during follow-up after the initial observation period in the CCP group compared with the control group (20% vs. 60%). This might suggest less pulmonary impairment in the CCP group during the extended follow-up period.

The frequency of long COVID-19 varies greatly in the literature and ranges up to a proportion of over 80% of patients who report at least 1 long COVID-19 symptom (26, 58, 59). Common symptoms of long COVID-19 are fatigue (98%), myalgias (87%), headache (83%), and dyspnea (88%) (COVERSCAN study data, based on patients with persistent symptoms) (58). Organs whose function may be impaired in long COVID-19 include lungs, heart, liver, kidneys, and nervous system (29, 33, 58, 60). The COVERSCAN study reported that 70% of patients with long COVID-19 symptoms still had impairment in at least one organ system at least 4 months after acute COVID-19 (58). In a large cohort study from Wuhan, China, patients reported mainly fatigue and muscle weakness (63%), sleep disturbance (26%), and anxiety and depression (23%) after a median time of 176 days (34). Pulmonary diffusion disorders were detectable during follow-up of 56% of patients with WHO grade 5 or 6 COVID-19 (34). A high proportion of patients also reported memory loss, concentration and sleep disturbances, and persistent loss of smell or taste (61–63). Other studies also report similar frequencies and variety of symptomatology as well as organ involvement in long COVID-19 (27–29, 33, 35, 59–67). A subgroup of patients had structural organ damage (lung, heart, and nervous systems, whereas the rest had functional complaints without organ damage [“functional long COVID-19”]) (68). Overall, the pattern of symptoms, their frequency, and severity in the long-term observation is consistent with reports on COVID-19 in the literature, but provides data on donors and patients in a randomized CCP trial.

The vast majority of both donors and patients were vaccinated and responded well to vaccination, while patients showed a significantly more pronounced increase in their antibody titers. At baseline, the amount of anti–SARS-CoV-2 IgG antibodies was comparable between the donors and the patients, but patients showed a substantially higher level of anti–SARS-CoV-2 IgA. This might reflect the different severity of COVID-19 in the patient and CCP donor population and the different timing of sampling. At baseline, CCP donors had recovered, while patients were in the acute phase of the infection. The higher antibody titers in patients compared with donors might be associated with the different severity of COVID-19. However, we cannot rule out the possibility that the difference is due to other confounding variables that might influence antibody levels, e.g., age, BMI, or a different timing of immunization events. Patients were significantly older and their interval since last vaccination and antibody measurement was longer than in donors (Table 1). There has been the concern that CCP treatment might impair response to vaccination later on (69). Our limited data set does not support this notion. This aspect needs further investigation as we continue to use and design antibody-based therapies for COVID-19 and other infectious diseases.

The main shortcoming of our study is the limited sample size, which included only 50 patients in the long-term follow-up. The CAPSID trial treated patients with severe COVID-19. More than 50% of patients included in this long-term observation period had a baseline WHO score of 5 or higher and the duration from symptom start to randomization was 8 (IQR, 5.0–11.0) days. Meanwhile, there are trials and registry studies that suggest a higher efficacy of CCP when it is given early in the course of COVID-19 to patients with mild symptoms (10, 18, 36, 70, 71). Therefore, the long-term effect of CCP might be too subtle in this small cohort that represents a subgroup of patients with poor prognosis due to advanced disease and late CCP treatment. The small sample size also limits a more detailed analysis of QoL and antibody responses in the subgroups treated with low or high amounts of neutralizing antibodies. Nevertheless, these data can provide a reference for the long-term burden of disease in patients treated in a CCP trial, in particular since several validated and internationally widely used QoL instruments have been used and a reference cohort of patients with mild to moderate disease (donors) was included.

In conclusion, the consistent trend for a benefit across several endpoints (OS, time to first negative SARS-CoV-2 PCR, discharge from ICU, discharge from hospital) among patients who received a larger cumulative amount of neutralizing antibodies is confirmed in the extended observation period. There was also a consistent trend for an improved QoL for patients treated with CCP across several dimensions by 3 different QoL instruments. Given the substantial long-term disease burden in some patients, the therapeutic long-term effects of CCP are of great interest and long-term observations shall be reported from CCP clinical trials conducted so far, and should in particular be further investigated in upcoming larger clinical trials that take into account the lessons learned so far regarding the selection of CCP units, dose, and timing of administration and the vulnerable patient population.

## Methods

### Design.

This is a long-term follow-up of the CAPSID trial, a multicenter, open-label randomized clinical trial to evaluate the efficacy and safety of CCP added to standard therapy (CCP group) versus standard therapy alone (control group) in hospitalized patients with COVID-19 (Figure 1). Patients in the CCP group received 3 units of plasma, with a median total volume of 846 mL. The CAPSID trial recruited 106 patients from 13 hospitals in Germany in the period from August 30, 2020 to December 24, 2020. The initial protocol included a follow-up for 60 days that was completed on February 23, 2021. Results of the first analysis of patients based on an interim data cutoff on April 28, 2021 and the analysis of donor and CCP characteristics have been published previously (1, 31). In a protocol amendment, a follow-up period up to 15 months was included for patients and CCP donors. The CCP donors were included as a reference group with asymptomatic to moderate disease for comparison of the burden of disease in the clinical trial patients. The objectives of the extended follow-up were to analyze long-term survival and frequency and severity of long COVID-19 in CCP donors and patients, to study the impact of CCP treatment and the CCP dose (in terms of cumulative amount of neutralizing antibodies) on long COVID-19 and long-term immunity.

### Patients and donors.

A total of 50 patients and 113 donors in 12 hospitals and 7 donor centers in Germany participated in the long-term follow-up between November 5, 2021 and February 19, 2022.

Inclusion criteria for the long-term follow were as follows: (a) patients who were enrolled in the CAPSID trial or recruited CCP donors for the CAPSID trial and (b) signed informed consent for the participation in the follow-up. Inclusion criteria for patients and donors were published recently (1, 31).

One outpatient visit between day 240 and 540 after randomization or first plasma donation was planned. The following assessments and data collections were performed: medical history including symptoms, complications, hospital treatments, medication, and chest imaging since the previous end of study, heart rate and blood pressure, QoL questionnaires (EQ-5D-5L, FACIT fatigue and FACIT Dyspnea), and blood tests for inflammation markers, coagulation markers, anti–SARS-CoV-2 immunity, and organ function. A structured interview was performed using a prespecified questionnaire and the long COVID scale (72).

Patients who could not visit the study center could also participate by telephone. In these cases, no laboratory values were collected and no functional tests were performed.

### SARS-CoV-2 antibody assays.

PRNT and ELISA for the detection of IgG and IgA against the S protein of SARS-CoV-2 were performed as previously described (1, 73–75).

### Outcome measures.

The outcome measures of the long-term follow-up were as follows: (a) long-term survival up to 18 months after randomization (patients in the CCP group compared to control group) or first plasma donation (CCP donors); (b) frequency, severity, and duration of long COVID-19 up to 18 months after randomization (patients in the CCP group compared to control group) or first plasma donation (CCP donors); (c) resolution of pneumonia and functional recovery in patients (CCP group compared to control group and donors); (d) fatigue, QoL, and utilization of health care resources; (e) anti–SARS-CoV-2 immunity and inflammation, the effect of SARS-CoV-2 vaccination. For all endpoints (a–e), subgroup analysis by the cumulative amount of transfused neutralizing units in the CCP was planned.

The 5Q-5D-5L questionnaire assesses 5 dimensions: mobility, self care, usual activities, pain/discomfort, anxiety in 5 categories and one’s health today by a visual analog scale giving an EQ-5D-5L index score (76). FACIT Fatigue and FACIT Dyspnea were also used. The FACIT Fatigue questionnaire, which consists of 13 questions, was originally developed to understand the impact of anemia and fatigue on the daily activities of cancer patients, but it has also been used for many other chronic diseases (77). For each question, there are 5 response options, depending on the severity, ranging from “not at all” to “frequently.” The total score is on a numerical scale from 0 to 52, whereby the higher the score, the less the fatigue.

The FACIT Dyspnea questionnaire consist of 10 questions and 10 ratings (78). It was originally developed to measure dyspnea severity and related functional limitations in patients with chronic obstructive pulmonary disease (COPD), but it has also been used for many other diseases (78, 79). Details of QoL questionnaires with questions are listed in Supplemental Tables 2–13. The post–COVID-19 scale grades the functional limitations from no functional limitations (grade 0) to severe functional limitations (grade 4) using 4 questions (72): (a) Can you live alone without any assistance from another person (e.g., independently being able to eat, walk, use the toilet, and manage routine daily hygiene)? (b) Are there any duties/activities at home or at work that you are no longer able to perform yourself? (c) Do you suffer from symptoms, pain, depression, or anxiety? (d) Do you need to avoid or reduce duties/activities or spread these over time? The complete algorithm is shown in Supplemental Figure 1.

Outcome measures for the primary and secondary outcome have been previously reported (1). Patients who died during the observation period without reaching the secondary outcome were censored as if they had reached the end of observation to account for the competing risk setting. The primary and secondary outcomes were also analyzed in a subgroup analysis by transfused neutralizing units. Since the total amount of neutralizing antibodies depends on both the volume and the antibody titer of CCP, we used “neutralizing units” to take into account both variables. One neutralizing unit was arbitrarily defined as 1 mL of CCP with a PRNT50 titer of 1:20. The neutralizing units of a CCP transfusion unit were then calculated by dividing the titer by 20 and multiplying by volume (mL) (1). The CCP group was divided by the cumulative amount of neutralizing units per patient (all 3 CCP transfusions) into a low neutralizing unit group (≤ median) and a high neutralizing unit group (> median).

Symptoms were documented and reported according to Common Terminology Criteria for Adverse Events (CTCAE version 4.0; https://evs.nci.nih.gov/ftp1/CTCAE/CTCAE_4.03/Archive/CTCAE_4.0_2009-05-29_QuickReference_8.5x11.pdf).

### Statistics.

All patients with long-term follow-up information and all participating donors were considered for analysis of OS. Unless otherwise stated, the quantitative results indicate the median of the respective group and the numerical values in brackets indicate the IQR.

Nominal and ordinal variables were analyzed using absolute frequencies and percentages. Missing values were considered as a separate category. Continuous variables like QoL or laboratory values including PRNT50 are described by reporting the median and IQR for the total number of patients and donors who provided values.

Secondary outcomes were analyzed using a Kaplan-Meier estimation procedure. Patients who died during observation without reaching the secondary outcome were censored as if they had reached the end of observation to account for competing risk. In prespecified subgroup analyses, outcomes were assessed in patients with low or high levels of neutralizing units (cumulative neutralizing units of all CCP products transfused equal to or below the median or above the median) and in subgroups created by the amount of donor symptoms with the corresponding CCP units.

An unpaired, 2-tailed Mann-Whitney test or a 2-tailed, paired Wilcoxon’s matched-pair test was used to analyze the continuous variables. A *P* value of less than 0.05 was considered to be statistically significant.

Statistical analyses were performed according to the statistical analysis plan using SAS (version 9.4M6 or newer; www.sas.com) or GraphPad Prism software version 9.0.3. The analysis for this manuscript was based on a final data cutoff of March 16, 2022.

### Study approval.

The trial was approved by the Federal Authority Paul-Ehrlich-Institute and by the Ethical Committee of the University of Ulm and the ethical committees of the participating hospitals. The trial is registered: EudraCT number 2020-001310-38 and ClinicalTrials.gov NCT04433910. Written informed consent was obtained from all study participants or their legal representatives.

## Author contributions

HS and SK wrote the study protocol, coordinated the study, analyzed and interpreted data, and wrote the manuscript. HS, SK, and ES provided funding. HS and ES were the lead investigators. BG, DZ, TW, KZ, P Spieth, PR, GP, T Thiele, JK, MMD, JK, MB, GSS, PML, LE, HW, MW, and JMK contributed clinical advice, patient enrollment, patient care, and data collection, including the extended observation period. PW, RB, T Tonn, JP, JS, T Bakchoul, DK, AU, HK, BJ, RL, T Burkhardt, RM, and MR contributed donor care, plasma collection, and data collection, including the long-term observation period. VMC and BJ analyzed SARS-CoV-2 antibodies. MS performed SARS-CoV-2 PCR. TA and MR provided project and sample management. BM and P Schnecko provided statistical advice. All authors have approved the final manuscript.

## Supplementary Material

Supplemental data

Trial reporting checklists

ICMJE disclosure forms

## Figures and Tables

**Table 1:**
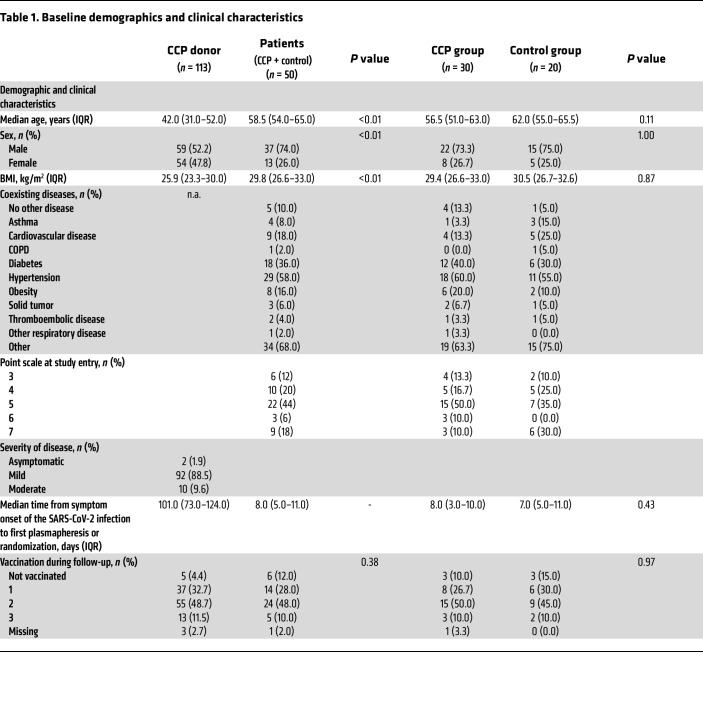
Baseline demographics and clinical characteristics

**Table 2:**
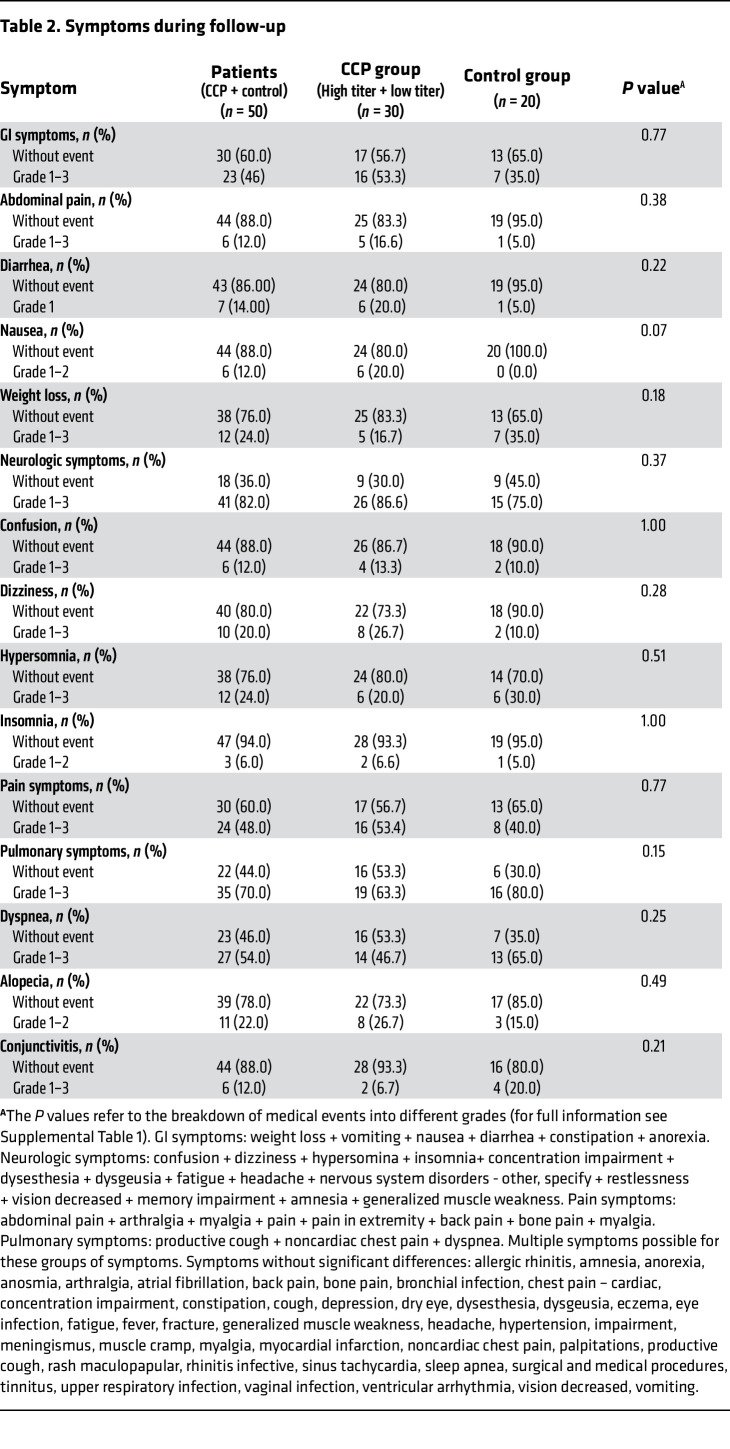
Symptoms during follow-up

**Table 3:**
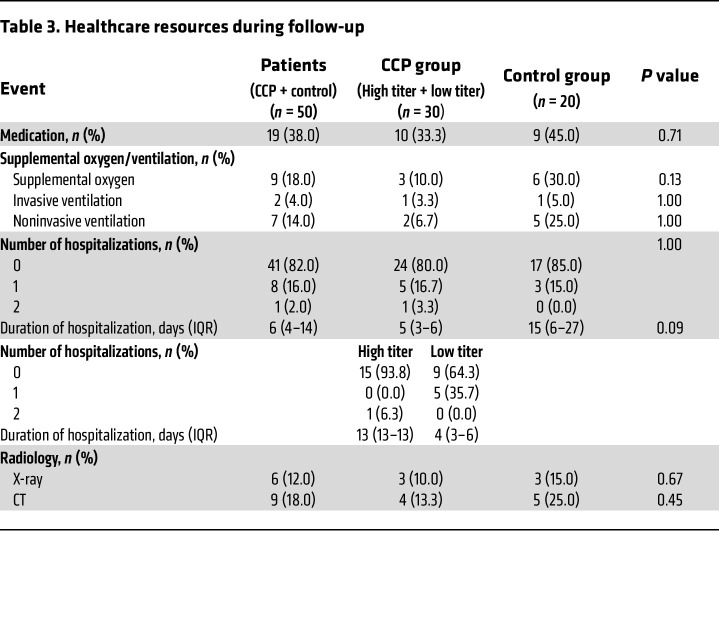
Healthcare resources during follow-up

## Version 1 11/03/2022

In-Press Preview

## Version 2 12/15/2022

Electronic publication
