# Supplementary material for: One-year follow-up of the CAPSID randomized trial for high-dose convalescent plasma in severe COVID-19 patients
Source: J Clin Invest. 2022 Dec 15;132(24):e163657. doi: 10.1172/JCI163657 (PMC9753994; doi:10.1172/JCI163657)
Supplement: Supplemental data [file jci-132-163657-s059.pdf]

Long-term outcome after COVID-19 convalescent plasma treatment in patients with severe COVID-19: One year follow up of the randomized clinical trial CAPSID

Supplement

Sixten Körper[1], Beate Grüner[2], Daniel Zickler[3], Thomas Wiesmann[4,5], Patrick Wuchter[6], Rainer Blasczyk[7], Kai Zacharowski[8], Peter Spieth[9] , Torsten Tonn[10] , Peter Rosenberger[11] , Gregor Paul[12], Jan Pilch[13], Joachim Schwäble[14], Tamam Bakchoul[15], Thomas Thiele[16] , Julian Knörlein[17], Matthias M. Dollinger[18] , Jörg Krebs[19], Martin Bentz[20], Victor M. Corman[21], Dzenan Kilalic[1], Gerlinde Schmidtke-Schrezenmeier[22], Philipp M. Lepper[23], Lucas Ernst[3], Hinnerk Wulf[4], Alexandra Ulrich[1], Manfred Weiss[24], Jan Matthias Kruse[3], Thomas Burkhardt[10], Rebecca Müller[6], Harald Klüter[6], Michael Schmidt[14], Bernd Jahrsdörfer[1], Ramin Lotfi[1] , Markus Rojewski[1], Thomas Appl[1], Benjamin Mayer[25], Philipp Schnecko[26], Erhard Seifried[14] , Hubert Schrezenmeier[1#]

[1] Institute for Clinical Transfusion Medicine and Immunogenetics Ulm, German Red Cross Blood Transfusion Service Baden-Württemberg-Hessen and University Hospital Ulm and Institute of Transfusion Medicine, University of Ulm.
[2] Division of Infectious Diseases, University Hospital and Medical Center Ulm, Ulm, Germany
[3] Department of Nephrology and Medical Intensive Care, Charité - Universitätsmedizin Berlin, corporate member of Free University Berlin, Humboldt-Universität zu Berlin, and Berlin Institute of Health, Berlin, Germany
[4] Department of Anaesthesiology and Intensive Care Medicine, Phillips-University Marburg, Marburg, Germany
[5] Present Address: Dept. of Anesthesiology, Critical Care Medicine & Pain Therapy, Diakonie Krankenhaus Schwäbisch Hall, Germany
[6] Institute of Transfusion Medicine and Immunology, German Red Cross Blood Transfusion Service Baden-Württemberg-Hessen, Medical Faculty Mannheim, Heidelberg University, Germany.
[7] Institute of Transfusion Medicine and Transplant Engineering, Hannover Medical School, Hannover, Geramny
[8] Department of Anaesthesiology, Intensive Care Medicine and Pain Therapy, University Hospital Frankfurt, Goethe-University, Germany
[9] Department of Anesthesiology and Critical Care Medicine, Carl Gustav Carus University Hospital, Technische Universität Dresden, Dresden, Germany
[10] Transfusion Medicine, Medical Faculty Carl Gustav Carus, Technische Universität Dresden and German Red Cross Blood Donation Service North-East gGmbH, Dresden, Germany
[11] Department of Anesthesiology and Intensive Care Medicine, University Hospital Tübingen, Tübingen, Germany
[12] Department of Gastroenterology, Hepatology, Pneumology and Infectious Diseases, Klinikum Stuttgart, Stuttgart, Germany
[13] Institute of Clinical Hemostaseology and Transfusion Medicine, Saarland University Hospital, Homburg/Saar, Germany
[14] Institute of Transfusion Medicine and Immunohematology, German Red Cross Blood Transfusion Service Baden-Württemberg – Hessen, Frankfurt, Germany
[15] Institute of Clinical and Experimental Transfusion Medicine, University Hospital Tübingen, Tübingen, Germany
[16] Institute of Transfusion Medicine, University Hospital Greifswald, Greifswald, Germany
[17] Clinic of Anesthesiology and Intensive Care Medicine University Medical Center of Freiburg, Germany
[18] Medical Clinic I, Klinikum Landshut, Landshut, Germany
[19] Clinic for Anesthesiology and Surgical Intensive Care Medicine, University of Mannheim, Mannheim, Germany
[20] Department of Internal Medicine III, Hospital of Karlsruhe, Karlsruhe, Germany
[21] Institute of Virology, Charité- University Medicine Berlin, corporate member of Free University Berlin, Humboldt-Universität zu Berlin, and Berlin Institute of Health and German Centre for Infection Research, Berlin, Germany
[22] Clinic of Internal Medicine II, Ulm University, Ulm Germany
[23] Department of Internal Medicine V – Pneumology, Allergology, Intensive Care Medicine, Saarland University Hospital, Homburg, Germany
[24] Department of Anaesthesiology and Intensive Care Medicine, University Hospital Ulm, Ulm University, Ulm, Germany
[25] Institute of Epidemiology and Medical Biometry, Ulm University, Ulm, Germany
[26] Alcedis GmbH, Gießen, Germany

Funding:

The clinical trial CAPSID including this long-term observation period is supported by Bundesministerium für Gesundheit ("German Federal Ministry of Health"): ZMVI1-2520COR802 and ZMI1-2521COR802

Gefördert durch:

Bundesministerium für Gesundheit

aufgrund eines Beschlusses des Deutschen Bundestages

Table of contents

Supplemental Figure 1. Post-COVID-19 Scale ... 3

Supplemental Figure 2: Course of Anti-SARS-CoV2 antibodies by ELISA ... 4

Table 1: Symptoms and health care resources during follow up ... 5

Table 2: EQ-5D-5L patients and donors ... 10

Table 3: EQ-5D-5L by randomization group ... 12

Table 4: EQ-5D-5L by transfused titer ... 14

Table 5: FACIT Dyspnea 10 Item Short Form – Part 1, donors and patients ... 16

Table 6: FACIT Dyspnea 10 Item Short Form – Part 1 by randomization group ... 19

Table 7: FACIT Dyspnea 10 Item Short Form – Part 1 by transfused titer ... 22

Table 8: FACIT Dyspnea 10 Item Short Form – Part 2 donors and patients ... 24

Table 9: FACIT Dyspnea 10 Item Short Form – Part 2 by randomization group ... 27

Table 10: FACIT Dyspnea 10 Item Short Form – Part 2 by transfused titer ... 30

Table 11: FACIT Fatigue Scale, donors and patients ... 32

Table 12: FACIT Fatigue Scale by randomization group ... 35

Table 13: FACIT Fatigue Scale by transfused titer ... 38

Table 14: Laboratory parameters CCP donors and trial patients ... 41

Table 15: Classification to assess disease severity of CCP donors. ... 42

Methods: Propensity score matching ... 43

Table 16 Propensity Score matching: Donor / Patients ... 43

Table 17 Medical events and Symptoms in propensity score matched cohort ... 44

Supplemental Figure 3: Post-COVID-19 Scale and change in socioeconomic status in the propensity score matched cohort ... 51

Supplemental Figure 4: QoL Scores of the propensity score matched cohort ... 52

Supplemental Figure 1. Post-COVID-19 Scale

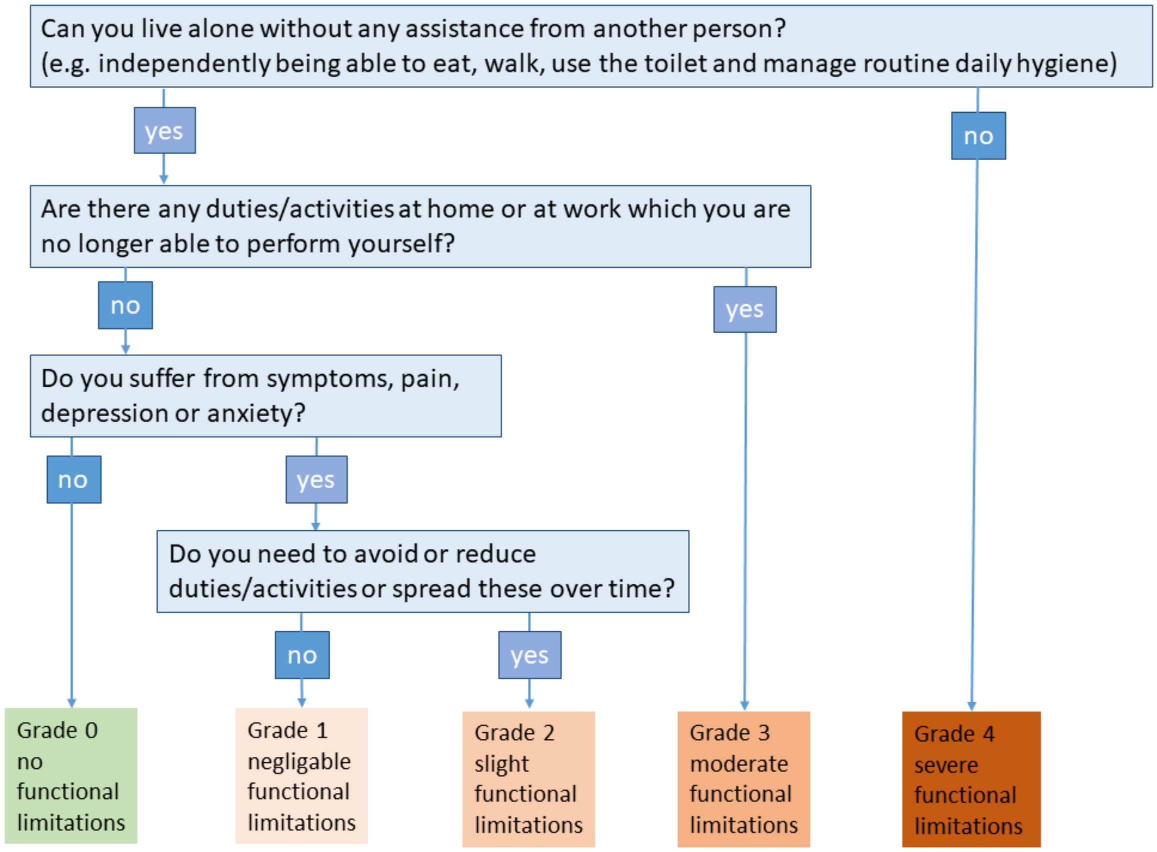

Supplemental Figure 2: Course of Anti-SARS-CoV2 antibodies by ELISA

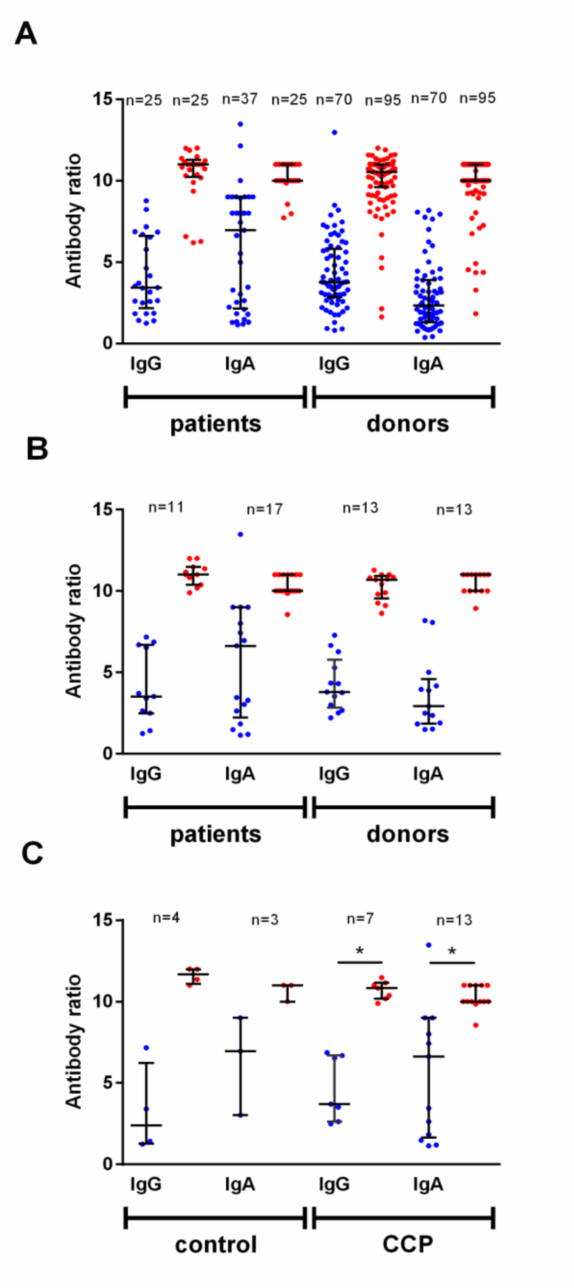

Supplemental Figure 2: Course of Anti-SARS-CoV2 antibodies by ELISA

Data are given as median and interquartile ranges. Blue dots represent baseline values und blue lines follow up data. Ratios are shown for IgG and IgA antibodies.

(A) All available follow up data and baseline data for participants are included. Donors IgG baseline vs. patients IgG baseline: p=0.5327. Donors IgG follow up vs. patients IgG follow up: P=0.036. Donors IgA baseline vs. patients IgA baseline: p<0.0001. Donors IgA follow up vs. patients IgA follow up: p=0.7209.

(B) Only vaccinated patients and donors with available baseline and follow up data are included.

(C) Only vaccinated patients with available baseline and follow up data are included. IgG (p=0.0156) and IgA (p=0.010) increased significantly after vaccination. No tests were performed for pairs with n < 7.

Comparisons were performed using Mann Whitney test.

Table 1: Symptoms and health care resources during follow up

Symptom	CCP-Donor (n=113)	Patients (CCP + Control) (n=50)	p-value	CCP group High titer + Low titer (n=30)	Control Group (n=20)	p-value
**GI-Symptoms, n (%) Multiple answers possible**			<0.01			0.77
Without Event	104 (92.0)	30 (60.0)		17 (56.7)	13 (65.0)	
Grade 1	8 (7.1)	19 (38.0)		12 (40.0)	7 (35.0)	
Grade 2	1 (0.9)	3 (6.0)		3 (10.0)	0 (0.0)	
Grade 3	1 (0.9)	1 (2.0)		1 (3.3)	0 (0.0)	
**GI-Symptoms, n (%) Worst grade**			<0.01			0.56
Without Event	104 (92.0)	30 (60.0)		17 (56.7)	13 (65.0)	
Grade 1	7 (6.2)	16 (32.0)		9 (30.0)	7 (35.0)	
Grade 2	1 (0.9)	3 (6.0)		3 (10.0)	0 (0.0)	
Grade 3	1 (0.9)	1 (2.0)		1 (3.3)	0 (0.0)	
**Abdominal pain, n (%)**			<0.01			0.38
Without Event	112 (99.1)	44 (88.0)		25 (83.3)	19 (95.0)	
Grade 1	1 (0.9)	3 (6.0)		3 (10.0)	0 (0.0)	
Grade 2	0 (0.0)	2 (4.0)		1 (3.3)	1 (5.0)	
Grade 3	0 (0.0)	1 (2.0)		1 (3.3)	0 (0.0)	
**Diarrhea, n (%)**			<0.01			0.22
Without Event	110 (97.4)	43 (86.00)		24 (80.0)	19 (95.0)	
Grade 1	3 (2.65)	7 (14.00)		6 (20.0)	1 (5.0)	
**Nausea, n (%)**			<0.01			0.07
Without Event	112 (99.1)	44 (88.0)		24 (80.0)	20 (100.0)	
Grade 1	0 (0.0)	5 (10.0)		5 (16.7)	0 (0.0)	
Grade 2	1 (0.9)	1 (2.0)		1 (3.3)	0 (0.0)	
**Weight loss, n (%)**			<0.01			0.18
Without Event	110 (97.4)	38 (76.0)		25 (83.3)	13 (65.0)	
Grade 1	3 (2.7)	9 (18.0)		2 (6.7)	7 (35.0)	
Grade 2	0 (0.0)	2 (4.0)		2 (6.7)	0 (0.0)	

Symptom	CCP-Donor (n=113)	Patients (CCP + Control) (n=50)	p-value	CCP group High titer + Low titer) (n=30)	Control Group (n=20)	p-value
Grade 3	0 (0.0)	1 (2.0)		1 (3.3)	0 (0.0)	
Neurologic-Symptoms, n (%) Multiple answers possible			0.49			0.37
Without Event	48 (42.5)	18 (36.0)		9 (30.0)	9 (45.0)	
Grade 1	61 (54.0)	24 (48.0)		18 (60.0)	6 (30.0)	
Grade 2	20 (17.7)	14 (28.0)		7 (23.3)	7 (35.0)	
Grade 3	4 (3.4)	3 (6.0)		1 (3.3)	2 (10.0)	
Neurologic-Symptoms, n (%) Worst grade			0.61			0.25
Without Event	48 (42.5)	18 (36.0)		9 (30.0)	9 (45.0)	
Grade 1	43 (38.1)	18 (36.0)		14 (46.7)	4 (20.0)	
Grade 2	18 (15.9)	11 (22.0)		6 (20.0)	5 (25.0)	
Grade 3	4 (3.5)	3 (6.0)		1 (3.3)	2 (10.0)	
Confusion, n (%)			0.01			1.00
Without Event	111 (98.2)	44 (88.0)		26 (86.7)	18 (90.0)	
Grade 1	2 (1.8)	3 (6.0)		3 (10.0)	0 (0.0)	
Grade 2	0 (0.0)	2 (4.0)		1 (3.3)	1 (5.0)	
Grade 3	0 (0.0)	1 (2.0)		0 (0.0)	1 (5.0)	
Dizziness, n (%)			0.03			0.28
Without Event	104 (92.0)	40 (80.0)		22 (73.3)	18 (90.0)	
Grade 1	7 (6.2)	9 (18.0)		8 (26.7)	1 (5.0)	
Grade 2	1 (0.9)	1 (2.0)		0 (0.0)	1 (5.0)	
Grade 3	1 (0.9)	0 (0.0)		0 (0.0)	0 (0.0)	
Hypersomnia, n (%)			0.03			0.51
Without Event	101 (89.4)	38 (76.0)		24 (80.0)	14 (70.0)	
Grade 1	8 (7.1)	4 (8.0)		3 (10.0)	1 (5.0)	
Grade 2	4 (3.5)	7 (14.0)		3 (10.0)	4 (20.0)	
Grade 3	0 (0.0)	1 (2.0)		0 (0.0)	1 (5.0)	
Insomnia, n (%)			0.03			1.00
Without Event	113 (100.0)	47 (94.0)		28 (93.3)	19 (95.0)	

Symptom	CCP-Donor (n=113)	Patients (CCP + Control) (n=50)	p-value	CCP group High titer + Low titer) (n=30)	Control Group (n=20)	p-value
Grade 1	0 (0.0)	1 (2.0)		1 (3.3)	0 (0.0)	
Grade 2	0 (0.0)	2 (4.0)		1 (3.3)	1 (5.0)	
**Pain-Symptoms, n (%) Multiple answers possible**			<0.01			0.77
Without Event	95 (84.1)	30 (60.0)		17 (56.7)	13 (65.0)	
Grade 1	12 (10.6)	14 (28.0)		8 (26.7)	6 (30.0)	
Grade 2	6 (5.3)	8 (16.0)		6 (20.0)	2 (10.0)	
Grade 3	1 (0.9)	2 (4.0)		2 (6.7)	0 (0.0)	
**Pain-Symptoms, n (%) Worst grade**			<0.01			0.73
Without Event	95 (84.1)	30 (60.0)		17 (56.7)	13 (65.0)	
Grade 1	11 (9.7)	11 (22.0)		6 (20.0)	5 (25.0)	
Grade 2	6 (5.3)	7 (14.0)		5 (16.7)	2 (10.0)	
Grade 3	1 (0.9)	2 (4.0)		2 (6.7)	0 (0.0)	
**Pulmonal Symptoms, n (%) Multiple answers possible**			0.03			0.15
Without Event	71 (62.8)*	22 (44.0)		16 (53.3)	6 (30.0)	
Grade 1	36 (31.9)	16 (32.0)		10 (33.3)	6 (30.0)	
Grade 2	8 (7.1)	15 (30.0)		7 (23.3)	8 (40.0)	
Grade 3	1 (0.9)	4 (8.0)		2 (6.7)	2 (10.0)	
**Pulmonal Symptoms, n (%) Worst grade**			<0.01			0.44
Without Event	71 (62.8)	22 (44.0)		16 (53.3)	6 (30.0)	
Grade 1	33 (29.2)	12 (24.0)		6 (20.0)	6 (30.0)	
Grade 2	8 (7.1)	12 (24.0)		6 (20.0)	6 (30.0)	
Grade 3	1 (0.9)	4 (8.0)		2 (6.7)	2 (10.0)	
**Dyspnea, n (%)**			<0.01			0.25
Without Event	84 (74.3)	23 (46.0)		16 (53.3)	7 (35.0)	
Grade 1	22 (19.5)	12 (24.0)		7 (23.3)	5 (25.0)	
Grade 2	6 (5.3)	12 (24.0)		5 (16.7)	7 (35.0)	

Symptom	CCP-Donor (n=113)	Patients (CCP + Control) (n=50)	p-value	CCP group High titer + Low titer) (n=30)	Control Group (n=20)	p-value
Grade 3	1 (0.9)	3 (6.0)		2 (6.7)	1 (5.0)	
Alopecia, n (%)			0.02			0.49
Without Event	105 (92.9)	39 (78.0)		22 (73.3)	17 (85.0)	
Grade 1	6 (5.3)	9 (18.0)		6 (20.0)	3 (15.0)	
Grade 2	2 (1.8)	2 (4.0)		2 (6.7)	0 (0.0)	
Conjuctivits, n (%)			0.01			0.21
Without Event	111 (98.2)	44 (88.0)		28 (93.3)	16 (80.0)	
Grade 1	2 (1.8)	4 (8.0)		2 (6.7)	2 (10.0)	
Grade 2	0 (0.0)	1 (2.0)		0 (0.0)	1 (5.0)	
Grade 3	0 (0.0)	1 (2.0)		0 (0.0)	1 (5.0)	
Medication, n (%)	7 (6.2)	19 (38.0)	<0.01	10 (33.3)	9 (45.0)	0.71
Supplemental Oxygen/Ventila-tion, n (%)						
Supplemental oxygen?	3 (2.7)	9 (18.0)	<0.01	3 (10.0)	6 (30.0)	0.13
Invasive ventilation	0 (0.0)	2 (4.0)	1.00	1 (3.3)	1 (5.0)	1.00
Non-invasive ventilation	2 (1.8)	7 (14.0)	1.00	2 (6.7)	5 (25.0)	1.00
Number of Hospitalisations, n (%)			0.053			1.0
0	105 (92.9)	41 (82.0)		24 (80.0)	17 (85.0)	
1	7 (6.2)	8 (16.0)		5 (16.7)	3 (15.0)	
2	0 (0.0)	1 (2.0)		1 (3.3)	0 (0.0)	
3	1 (0.9)	0 (0.0)		0 (0.0)	0 (0.0)	
Duration of Hospitalisations, days (IQR)	2 (2-2)	6 (4-14)	1.00	5 (3-6)	15 (6-27)	0.09
Number of Hospitalisations, n (%)				High titer	Low titer	
0				15 (93.8)	9 (64.3)	0.01
1				0 (0.0)	5 (35.7)	
2				1 (6.3)	0 (0.0)	
Duration of Hospitalisations, days (IQR)				13 (13-13)	4 (3 – 6)	

Symptom	CCP-Donor (n=113)	Patients (CCP + Control) (n=50)	p-value	CCP group High titer + Low titer) (n=30)	Control Group (n=20)	p-value
**Radiology, n (%)**						
X-Ray	18 (15.9)	6 (12.0)	0.63	3 (10.0)	3 (15.0)	0.67
CT	6 (5.3)	9 (18.0)	0.02	4 (13.3)	5 (25.0)	0.45

1. Pain-Symptoms: Abdominal Pain + Arthralgia+ Myalgia + Pain + Pain in extremity + Back pain + Bone pain + Myalgia

2. Neurologic: Confusion + Dizziness + Hypersomina + Insomnia+ Concentration impairment + Dysesthesia + Dysgeusia + Fatigue + Headache + Nervous system disorders - Other, specify + Restlessness + Vision decreased + Memory impairment + Amnesia + Generalized muscle weakness

3. GI-Symptoms: Weight loss + Vomiting + Nausea + Diarrhea + Constipation + Anorexia

4. Pulmonal Symptoms: Productive cough + Non-cardiac chest pain + Dyspnea

*Symptoms without significant differences: allergic rhinitis, amnesia, anorexia, anosmia, arthralgia, atrial fibrillation, back pain, bone pain, bronchial infection, chest pain – cardiac, concentration impairment, constipation, cough, depression, dry eye, dysaesthesia, dysgeusia, eczema, eye infection, fatigue, fever, fracture, generalized muscle weakness, headache, hypertension, impairment, meningismus, muscle cramp, myalgia, myocardial infarction, Non-cardiac chest pain, palpitations, productive cough, rash maculo-papular, rhinitis infective, sinus tachycardia, sleep apnea, surgical and medical procedures, tinnitus, upper respiratory infection, vaginal infection, Ventricular arrhythmia, vision decreased, vomiting

Table 2: EQ-5D-5L patients and donors

EQ-5D-5L	Donors	Patients	p-Value
Number of patients	113 (100.00)	50 (100.00)	
**Mobility**			<.0001
I have no problems in walking about	97 (85.84)	27 (54.00)	
I have slight problems in walking about	6 (5.31)	10 (20.00)	
I have moderate problems in walking about	3 (2.65)	6 (12.00)	
I have severe problems in walking about	0 (0.00)	4 (8.00)	
Not answered	1 (0.88)	0 (0.00)	
Missing	6 (5.31)	3 (6.00)	
**Self Care**			<.0001
I have no problems washing or dressing myself	103 (91.15)	34 (68.00)	
I have slight problems washing or dressing myself	3 (2.65)	7 (14.00)	
I have moderate problems washing or dressing myself	0 (0.00)	4 (8.00)	
I have severe problems washing or dressing my-self	0 (0.00)	1 (2.00)	
I am unable to wash or dress myself	0 (0.00)	1 (2.00)	
Not answered	1 (0.88)	0 (0.00)	
Missing	6 (5.31)	3 (6.00)	
**Usual Activities**			<.0001
I have no problems doing my usual activities	85 (75.22)	25 (50.00)	
I have slight problems doing my usual activities	16 (14.16)	8 (16.00)	
I have moderate problems doing my usual activi-ties	4 (3.54)	9 (18.00)	
I have severe problems doing my usual activities	0 (0.00)	4 (8.00)	
I am unable to do my usual activities	0 (0.00)	1 (2.00)	
Not answered	2 (1.77)	0 (0.00)	
Missing	6 (5.31)	3 (6.00)	
**Pain/Discomfort**			0.0007
I have no pain or discomfort	74 (65.49)	18 (36.00)	
I have slight pain or discomfort	21 (18.58)	16 (32.00)	
I have moderate pain or discomfort	9 (7.96)	6 (12.00)	
I have severe pain or discomfort	2 (1.77)	6 (12.00)	
I have extreme pain or discomfort	0 (0.00)	1 (2.00)	
Not answered	1 (0.88)	0 (0.00)	
Missing	6 (5.31)	3 (6.00)	

EQ-5D-5L	Donors	Patients	p-Value
Anxiety			**0.0300**
I am not anxious or depressed	76 (67.26)	28 (56.00)	
I am slightly anxious or depressed	23 (20.35)	9 (18.00)	
I am moderately anxious or depressed	6 (5.31)	4 (8.00)	
I am severely anxious or depressed	1 (0.88)	4 (8.00)	
I am extremely anxious or depressed	0 (0.00)	2 (4.00)	
Not answered	1 (0.88)	0 (0.00)	
Missing	6 (5.31)	3 (6.00)	
'Your health today' (VAS Score)			<.0001
N	107	46	
Mean	86.06	68.20	
STD	10.92	23.50	
Min	40.00	10.00	
Q1	80.00	50.00	
Median	90.00	70.00	
Q3	95.00	90.00	
Max	100.00	100.00	
NMiss	6	4	
EQ-5D-5L index score			**<.0001**
N	105	47	
Mean	0.96	0.80	
STD	0.08	0.24	
Min	0.47	0.19	
Q1	0.91	0.72	
Median	1.00	0.91	
Q3	1.00	1.00	
Max	1.00	1.00	
NMiss	8	3	

Table 3: EQ-5D-5L by randomization group

EQ-5D-5L	CCP	Control	p-Value
Number of patients in EFAS	30 (100.00)	20 (100.00)	
**Mobility**			**0.0395**
I have no problems in walking about	19 (63.33)	8 (40.00)	
I have slight problems in walking about	3 (10.00)	7 (35.00)	
I have moderate problems in walking about	5 (16.67)	1 (5.00)	
I have severe problems in walking about	1 (3.33)	3 (15.00)	
Missing	2 (6.67)	1 (5.00)	
Self Care			0.2345
I have no problems washing or dressing myself	23 (76.67)	11 (55.00)	
I have slight problems washing or dressing myself	3 (10.00)	4 (20.00)	
I have moderate problems washing or dressing myself	2 (6.67)	2 (10.00)	
I have severe problems washing or dressing myself	0 (0.00)	1 (5.00)	
I am unable to wash or dress myself	0 (0.00)	1 (5.00)	
Missing	2 (6.67)	1 (5.00)	
Usual Activities			0.3854
I have no problems doing my usual activities	17 (56.67)	8 (40.00)	
I have slight problems doing my usual activities	5 (16.67)	3 (15.00)	
I have moderate problems doing my usual activities	5 (16.67)	4 (20.00)	
I have severe problems doing my usual activities	1 (3.33)	3 (15.00)	
I am unable to do my usual activities	0 (0.00)	1 (5.00)	
Missing	2 (6.67)	1 (5.00)	
Pain/Discomfort			0.5729
I have no pain or discomfort	13 (43.33)	5 (25.00)	
I have slight pain or discomfort	8 (26.67)	8 (40.00)	
I have moderate pain or discomfort	3 (10.00)	3 (15.00)	
I have severe pain or discomfort	3 (10.00)	3 (15.00)	
I have extreme pain or discomfort	1 (3.33)	0 (0.00)	
Missing	2 (6.67)	1 (5.00)	

EQ-5D-5L	CCP	Control	p-Value
Anxiety			1.0000
I am not anxious or depressed	17 (56.67)	11 (55.00)	
I am slightly anxious or depressed	5 (16.67)	4 (20.00)	
I am moderately anxious or depressed	2 (6.67)	2 (10.00)	
I am severely anxious or depressed	3 (10.00)	1 (5.00)	
I am extremely anxious or depressed	1 (3.33)	1 (5.00)	
Missing	2 (6.67)	1 (5.00)	
'Your health today' (VAS Score)			0.2796
N	27	19	
Mean	71.37	63.68	
STD	21.97	25.43	
Min	10.00	10.00	
Q1	50.00	50.00	
Median	75.00	65.00	
Q3	90.00	90.00	
Max	100.00	100.00	
NMiss	3	1	
EQ-5D-5L index score			0.3545
N	28	19	
Mean	0.83	0.76	
STD	0.23	0.25	
Min	0.28	0.19	
Q1	0.66	0.72	
Median	0.91	0.83	
Q3	1.00	0.91	
Max	1.00	1.00	
NMiss	2	1	

Table 4: EQ-5D-5L by transfused titer

EQ-5D-5L	Low titer Plasma	High titer Plasma	p-Value
Number of patients in EFAS	14 (100.00)	16 (100.00)	
Mobility			0.6853
I have no problems in walking about	7 (50.00)	12 (75.00)	
I have slight problems in walking about	2 (14.29)	1 (6.25)	
I have moderate problems in walking about	3 (21.43)	2 (12.50)	
I have severe problems in walking about	0 (0.00)	1 (6.25)	
Missing	2 (14.29)	0 (0.00)	
Self Care			1.0000
I have no problems washing or dressing myself	10 (71.43)	13 (81.25)	
I have slight problems washing or dressing myself	1 (7.14)	2 (12.50)	
I have moderate problems washing or dressing my-self	1 (7.14)	1 (6.25)	
Missing	2 (14.29)	0 (0.00)	
Usual Activities			0.3026
I have no problems doing my usual activities	6 (42.86)	11 (68.75)	
I have slight problems doing my usual activities	2 (14.29)	3 (18.75)	
I have moderate problems doing my usual activities	4 (28.57)	1 (6.25)	
I have severe problems doing my usual activities	0 (0.00)	1 (6.25)	
Missing	2 (14.29)	0 (0.00)	
Pain/Discomfort			0.6411
I have no pain or discomfort	6 (42.86)	7 (43.75)	
I have slight pain or discomfort	2 (14.29)	6 (37.50)	
I have moderate pain or discomfort	2 (14.29)	1 (6.25)	
I have severe pain or discomfort	1 (7.14)	2 (12.50)	
I have extreme pain or discomfort	1 (7.14)	0 (0.00)	
Missing	2 (14.29)	0 (0.00)	
Anxiety			0.9521
I am not anxious or depressed	7 (50.00)	10 (62.50)	
I am slightly anxious or depressed	2 (14.29)	3 (18.75)	
I am moderately anxious or depressed	1 (7.14)	1 (6.25)	
I am severely anxious or depressed	2 (14.29)	1 (6.25)	
I am extremely anxious or depressed	0 (0.00)	1 (6.25)	
Missing	2 (14.29)	0 (0.00)	

EQ-5D-5L	Low titer Plasma	High titer Plasma	p-Value
'Your health today' (VAS Score)			0.7020
N	11	16	
Mean	69.36	72.75	
STD	26.14	19.40	
Min	10.00	40.00	
Q1	50.00	56.00	
Median	75.00	75.00	
Q3	90.00	90.00	
Max	100.00	99.00	
NMiss	3	0	
EQ-5D-5L index score			0.7297
N	12	16	
Mean	0.81	0.84	
STD	0.26	0.20	
Min	0.28	0.40	
Q1	0.66	0.71	
Median	0.91	0.91	
Q3	1.00	1.00	
Max	1.00	1.00	
NMiss	2	0	

Table 5: FACIT Dyspnea 10 Item Short Form – Part 1, donors and patients

FACIT Dyspnea 10	Donor	Patients	p-Value
Number of patients	113 (100.00)	50 (100.00)	
Dressing yourself without help			<.0001
No shortness of breath	102 (90.27)	30 (60.00)	
Mildly short of breath	5 (4.42)	11 (22.00)	
Moderately short of breath	0 (0.00)	6 (12.00)	
I did not do this in the past 7 days	0 (0.00)	1 (2.00)	
Missing	6 (5.31)	2 (4.00)	
Walking 50 steps on flat ground at a normal speed without stopping			<.0001
No shortness of breath	101 (89.38)	31 (62.00)	
Mildly short of breath	5 (4.42)	5 (10.00)	
Moderately short of breath	1 (0.88)	7 (14.00)	
Severely short of breath	0 (0.00)	3 (6.00)	
I did not do this in the past 7 days	0 (0.00)	2 (4.00)	
Missing	6 (5.31)	2 (4.00)	
Walking 20 stairs without stopping			0.0003
No shortness of breath	78 (69.03)	19 (38.00)	
Mildly short of breath	21 (18.58)	16 (32.00)	
Moderately short of breath	5 (4.42)	5 (10.00)	
Severely short of breath	2 (1.77)	5 (10.00)	
I did not do this in the past 7 days	0 (0.00)	2 (4.00)	
Missing	7 (6.19)	3 (6.00)	
Preparing meals			0.0005
No shortness of breath	105 (92.92)	41 (82.00)	
Mildly short of breath	0 (0.00)	5 (10.00)	
Moderately short of breath	1 (0.88)	1 (2.00)	
I did not do this in the past 7 days	0 (0.00)	1 (2.00)	
Missing	7 (6.19)	2 (4.00)	
Washing dishes			0.0001
No shortness of breath	103 (91.15)	35 (70.00)	
Mildly short of breath	2 (1.77)	7 (14.00)	
Moderately short of breath	0 (0.00)	2 (4.00)	
I did not do this in the past 7 days	2 (1.77)	4 (8.00)	
Missing	6 (5.31)	2 (4.00)	

FACIT Dyspnea 10	Donor	Patients	p-Value
**Sweeping or mopping**			<.0001
No shortness of breath	95 (84.07)	25 (50.00)	
Mildly short of breath	7 (6.19)	12 (24.00)	
Moderately short of breath	1 (0.88)	6 (12.00)	
Severely short of breath	1 (0.88)	1 (2.00)	
I did not do this in the past 7 days	3 (2.65)	4 (8.00)	
Missing	6 (5.31)	2 (4.00)	
**Making a bed**			<.0001
No shortness of breath	96 (84.96)	27 (54.00)	
Mildly short of breath	4 (3.54)	9 (18.00)	
Moderately short of breath	2 (1.77)	7 (14.00)	
I did not do this in the past 7 days	4 (3.54)	4 (8.00)	
Missing	7 (6.19)	3 (6.00)	
**Lifting something weighing 10-20 lbs**			<.0001
No shortness of breath	93 (82.30)	21 (42.00)	
Mildly short of breath	8 (7.08)	16 (32.00)	
Moderately short of breath	3 (2.65)	5 (10.00)	
Severely short of breath	2 (1.77)	3 (6.00)	
I did not do this in the past 7 days	1 (0.88)	3 (6.00)	
Missing	6 (5.31)	2 (4.00)	
**Carrying something weighing 10-20 lbs from one room to another**			<.0001
No shortness of breath	94 (83.19)	21 (42.00)	
Mildly short of breath	10 (8.85)	18 (36.00)	
Moderately short of breath	2 (1.77)	2 (4.00)	
Severely short of breath	1 (0.88)	3 (6.00)	
I did not do this in the past 7 days	0 (0.00)	4 (8.00)	
Missing	6 (5.31)	2 (4.00)	
**Walking for 1/2 a mile without stopping**			<.0001
No shortness of breath	72 (63.72)	15 (30.00)	
Mildly short of breath	25 (22.12)	11 (22.00)	
Moderately short of breath	7 (6.19)	10 (20.00)	
Severely short of breath	3 (2.65)	4 (8.00)	
I did not do this in the past 7 days	0 (0.00)	8 (16.00)	
Missing	6 (5.31)	2 (4.00)	

FACIT Dyspnea 10	Donor	Patients	p-Value
**Raw dyspnea score**			<.0001
**N**	107	48	
**Mean**	1.50	6.51	
**STD**	3.32	7.50	
**Min**	0.00	0.00	
**Q1**	0.00	0.00	
**Median**	0.00	4.00	
**Q3**	2.00	9.50	
**Max**	23.00	26.67	
**NMiss**	6	2	

Table 6: FACIT Dyspnea 10 Item Short Form – Part 1 by randomization group

FACIT Dyspnea 10	CCP	Control	p-Value
Number of patients in EFAS	30 (100.00)	20 (100.00)	
Dressing yourself without help			0.1678
No shortness of breath	21 (70.00)	9 (45.00)	
Mildly short of breath	6 (20.00)	5 (25.00)	
Moderately short of breath	2 (6.67)	4 (20.00)	
I did not do this in the past 7 days	0 (0.00)	1 (5.00)	
Missing	1 (3.33)	1 (5.00)	
Walking 50 steps on flat ground at a normal speed without stopping			0.1540
No shortness of breath	22 (73.33)	9 (45.00)	
Mildly short of breath	2 (6.67)	3 (15.00)	
Moderately short of breath	4 (13.33)	3 (15.00)	
Severely short of breath	1 (3.33)	2 (10.00)	
I did not do this in the past 7 days	0 (0.00)	2 (10.00)	
Missing	1 (3.33)	1 (5.00)	
Walking 20 stairs without stopping			0.4885
No shortness of breath	14 (46.67)	5 (25.00)	
Mildly short of breath	8 (26.67)	8 (40.00)	
Moderately short of breath	3 (10.00)	2 (10.00)	
Severely short of breath	2 (6.67)	3 (15.00)	
I did not do this in the past 7 days	1 (3.33)	1 (5.00)	
Missing	2 (6.67)	1 (5.00)	
Preparing meals			0.1723
No shortness of breath	27 (90.00)	14 (70.00)	
Mildly short of breath	2 (6.67)	3 (15.00)	
Moderately short of breath	0 (0.00)	1 (5.00)	
I did not do this in the past 7 days	0 (0.00)	1 (5.00)	
Missing	1 (3.33)	1 (5.00)	
Washing dishes			0.0892
No shortness of breath	24 (80.00)	11 (55.00)	
Mildly short of breath	4 (13.33)	3 (15.00)	
Moderately short of breath	0 (0.00)	2 (10.00)	
I did not do this in the past 7 days	1 (3.33)	3 (15.00)	
Missing	1 (3.33)	1 (5.00)	

FACIT Dyspnea 10	CCP	Control	p-Value
Sweeping or mopping			0.0381
No shortness of breath	17 (56.67)	8 (40.00)	
Mildly short of breath	7 (23.33)	5 (25.00)	
Moderately short of breath	5 (16.67)	1 (5.00)	
Severely short of breath	0 (0.00)	1 (5.00)	
I did not do this in the past 7 days	0 (0.00)	4 (20.00)	
Missing	1 (3.33)	1 (5.00)	
Making a bed			0.5760
No shortness of breath	17 (56.67)	10 (50.00)	
Mildly short of breath	6 (20.00)	3 (15.00)	
Moderately short of breath	4 (13.33)	3 (15.00)	
I did not do this in the past 7 days	1 (3.33)	3 (15.00)	
Missing	2 (6.67)	1 (5.00)	
Lifting something weighing 10-20 lbs			0.4514
No shortness of breath	14 (46.67)	7 (35.00)	
Mildly short of breath	11 (36.67)	5 (25.00)	
Moderately short of breath	2 (6.67)	3 (15.00)	
Severely short of breath	1 (3.33)	2 (10.00)	
I did not do this in the past 7 days	1 (3.33)	2 (10.00)	
Missing	1 (3.33)	1 (5.00)	
Carrying something weighing 10-20 lbs from one room to another			0.5457
No shortness of breath	13 (43.33)	8 (40.00)	
Mildly short of breath	12 (40.00)	6 (30.00)	
Moderately short of breath	0 (0.00)	2 (10.00)	
Severely short of breath	2 (6.67)	1 (5.00)	
I did not do this in the past 7 days	2 (6.67)	2 (10.00)	
Missing	1 (3.33)	1 (5.00)	
Walking for 1/2 a mile without stopping			0.7210
No shortness of breath	10 (33.33)	5 (25.00)	
Mildly short of breath	7 (23.33)	4 (20.00)	
Moderately short of breath	6 (20.00)	4 (20.00)	
Severely short of breath	1 (3.33)	3 (15.00)	
I did not do this in the past 7 days	5 (16.67)	3 (15.00)	
Missing	1 (3.33)	1 (5.00)	

FACIT Dyspnea 10	CCP	Control	p-Value
Raw dyspnea score			0.1959
N	29	19	
Mean	5.36	8.25	
STD	6.32	8.91	
Min	0.00	0.00	
Q1	0.00	0.00	
Median	3.33	6.00	
Q3	7.50	13.00	
Max	20.00	26.67	
NMiss	1	1	

Table 7: FACIT Dyspnea 10 Item Short Form – Part 1 by transfused titer

FACIT Dyspnea 10	Low titer Plasma	High titer Plasma	p-Value
Number of patients in EFAS	14 (100.00)	16 (100.00)	
Dressing yourself without help			0.3990
No shortness of breath	9 (64.29)	12 (75.00)	
Mildly short of breath	2 (14.29)	4 (25.00)	
Moderately short of breath	2 (14.29)	0 (0.00)	
Missing	1 (7.14)	0 (0.00)	
Walking 50 steps on flat ground at a normal speed without stopping			0.3746
No shortness of breath	10 (71.43)	12 (75.00)	
Mildly short of breath	0 (0.00)	2 (12.50)	
Moderately short of breath	3 (21.43)	1 (6.25)	
Severely short of breath	0 (0.00)	1 (6.25)	
Missing	1 (7.14)	0 (0.00)	
Walking 20 stairs without stopping			0.8543
No shortness of breath	5 (35.71)	9 (56.25)	
Mildly short of breath	4 (28.57)	4 (25.00)	
Moderately short of breath	2 (14.29)	1 (6.25)	
Severely short of breath	1 (7.14)	1 (6.25)	
I did not do this in the past 7 days	0 (0.00)	1 (6.25)	
Missing	2 (14.29)	0 (0.00)	
Preparing meals			0.1921
No shortness of breath	11 (78.57)	16 (100.00)	
Mildly short of breath	2 (14.29)	0 (0.00)	
Missing	1 (7.14)	0 (0.00)	
Washing dishes			0.2994
No shortness of breath	10 (71.43)	14 (87.50)	
Mildly short of breath	3 (21.43)	1 (6.25)	
I did not do this in the past 7 days	0 (0.00)	1 (6.25)	
Missing	1 (7.14)	0 (0.00)	
Sweeping or mopping			0.8746
No shortness of breath	7 (50.00)	10 (62.50)	
Mildly short of breath	3 (21.43)	4 (25.00)	
Moderately short of breath	3 (21.43)	2 (12.50)	
Missing	1 (7.14)	0 (0.00)	

FACIT Dyspnea 10	Low titer Plasma	High titer Plasma	p-Value
**Making a bed**			0.7874
No shortness of breath	7 (50.00)	10 (62.50)	
Mildly short of breath	2 (14.29)	4 (25.00)	
Moderately short of breath	2 (14.29)	2 (12.50)	
I did not do this in the past 7 days	1 (7.14)	0 (0.00)	
Missing	2 (14.29)	0 (0.00)	
**Lifting something weighing 10-20 lbs**			0.7639
No shortness of breath	6 (42.86)	8 (50.00)	
Mildly short of breath	4 (28.57)	7 (43.75)	
Moderately short of breath	1 (7.14)	1 (6.25)	
Severely short of breath	1 (7.14)	0 (0.00)	
I did not do this in the past 7 days	1 (7.14)	0 (0.00)	
Missing	1 (7.14)	0 (0.00)	
**Carrying something weighing 10-20 lbs from one room to another**	5 (35.71)	8 (50.00)	0.5928
No shortness of breath	5 (35.71)	7 (43.75)	
Mildly short of breath	2 (14.29)	0 (0.00)	
Severely short of breath	1 (7.14)	1 (6.25)	
I did not do this in the past 7 days	1 (7.14)	0 (0.00)	
Missing			
**Walking for 1/2 a mile without stopping**			0.8505
No shortness of breath	4 (28.57)	6 (37.50)	
Mildly short of breath	4 (28.57)	3 (18.75)	
Moderately short of breath	2 (14.29)	4 (25.00)	
Severely short of breath	1 (7.14)	0 (0.00)	
I did not do this in the past 7 days	2 (14.29)	3 (18.75)	
Missing	1 (7.14)	0 (0.00)	
**Raw dyspnea score**			0.5176
N	13	16	
Mean	6.23	4.66	
STD	7.18	5.68	
Min	0.00	0.00	
Q1	1.11	0.00	
Median	3.33	3.00	
Q3	9.00	7.25	
Max	20.00	20.00	
NMiss	1	0	

Table 8: FACIT Dyspnea 10 Item Short Form – Part 2 donors and patients

FACIT Dyspnea 10	Donors	Patients	p-Value
Number of patients	113 (100.00)	50 (100.00)	
Dressing yourself without help			<.0001
No difficulty	105 (92.92)	34 (68.00)	
A little difficulty	1 (0.88)	7 (14.00)	
Some difficulty	1 (0.88)	3 (6.00)	
Much difficulty	0 (0.00)	2 (4.00)	
Missing	6 (5.31)	4 (8.00)	
Walking 50 steps on flat ground at a normal speed without stopping			<.0001
No difficulty	105 (92.92)	32 (64.00)	
A little difficulty	1 (0.88)	4 (8.00)	
Some difficulty	0 (0.00)	7 (14.00)	
Much difficulty	1 (0.88)	2 (4.00)	
Missing	6 (5.31)	5 (10.00)	
Walking 20 stairs without stopping			<.0001
No difficulty	85 (75.22)	20 (40.00)	
A little difficulty	19 (16.81)	15 (30.00)	
Some difficulty	2 (1.77)	8 (16.00)	
Much difficulty	1 (0.88)	3 (6.00)	
Missing	6 (5.31)	4 (8.00)	
Preparing meals			0.0042
No difficulty	106 (93.81)	40 (80.00)	
A little difficulty	1 (0.88)	3 (6.00)	
Some difficulty	0 (0.00)	2 (4.00)	
Much difficulty	0 (0.00)	1 (2.00)	
Missing	6 (5.31)	4 (8.00)	
Washing dishes			0.0036
No difficulty	105 (92.92)	36 (72.00)	
A little difficulty	2 (1.77)	5 (10.00)	
Some difficulty	0 (0.00)	1 (2.00)	
Much difficulty	0 (0.00)	1 (2.00)	
Missing	6 (5.31)	7 (14.00)	

FACIT Dyspnea 10	Donors	Patients	p-Value
**Sweeping or mopping**			<.0001
No difficulty	99 (87.61)	27 (54.00)	
A little difficulty	6 (5.31)	10 (20.00)	
Some difficulty	0 (0.00)	4 (8.00)	
Much difficulty	1 (0.88)	2 (4.00)	
Missing	7 (6.19)	7 (14.00)	
**Making a bed**			<.0001
No difficulty	104 (92.04)	30 (60.00)	
A little difficulty	2 (1.77)	10 (20.00)	
Some difficulty	0 (0.00)	4 (8.00)	
Much difficulty	0 (0.00)	1 (2.00)	
Missing	7 (6.19)	5 (10.00)	
**Lifting something weighing 10-20 lbs**			<.0001
No difficulty	96 (84.96)	24 (48.00)	
A little difficulty	9 (7.96)	11 (22.00)	
Some difficulty	0 (0.00)	8 (16.00)	
Much difficulty	1 (0.88)	3 (6.00)	
Missing	7 (6.19)	4 (8.00)	
**Carrying something weighing 10-20 lbs from one room to another**			<.0001
No difficulty	91 (80.53)	21 (42.00)	
A little difficulty	12 (10.62)	12 (24.00)	
Some difficulty	2 (1.77)	11 (22.00)	
Much difficulty	1 (0.88)	2 (4.00)	
Missing	7 (6.19)	4 (8.00)	
**Walking for 1/2 a mile without stopping**			<.0001
No difficulty	76 (67.26)	17 (34.00)	
A little difficulty	26 (23.01)	10 (20.00)	
Some difficulty	2 (1.77)	9 (18.00)	
Much difficulty	2 (1.77)	7 (14.00)	
Missing	7 (6.19)	7 (14.00)	

FACIT Dyspnea 10	Donors	Patients	p-Value
**Raw functional limitation score**			<.0001
**N**	107	46	
**Mean**	1.07	6.08	
**STD**	2.69	7.46	
**Min**	0.00	0.00	
**Q1**	0.00	0.00	
**Median**	0.00	3.50	
**Q3**	1.00	9.00	
**Max**	22.00	29.00	
**NMiss**	6	4	

Table 9: FACIT Dyspnea 10 Item Short Form – Part 2 by randomization group

FACIT Dyspnea 10	CCP	Control	p-Value
Number of patients in EFAS	30 (100.00)	20 (100.00)	
Dressing yourself without help			0.1886
No difficulty	23 (76.67)	11 (55.00)	
A little difficulty	4 (13.33)	3 (15.00)	
Some difficulty	1 (3.33)	2 (10.00)	
Much difficulty	0 (0.00)	2 (10.00)	
Missing	2 (6.67)	2 (10.00)	
Walking 50 steps on flat ground at a normal speed without stopping			0.3318
No difficulty	21 (70.00)	11 (55.00)	
A little difficulty	2 (6.67)	2 (10.00)	
Some difficulty	4 (13.33)	3 (15.00)	
Much difficulty	0 (0.00)	2 (10.00)	
Missing	3 (10.00)	2 (10.00)	
Walking 20 stairs without stopping			0.5838
No difficulty	14 (46.67)	6 (30.00)	
A little difficulty	8 (26.67)	7 (35.00)	
Some difficulty	5 (16.67)	3 (15.00)	
Much difficulty	1 (3.33)	2 (10.00)	
Missing	2 (6.67)	2 (10.00)	
Preparing meals			0.1525
No difficulty	26 (86.67)	14 (70.00)	
A little difficulty	2 (6.67)	1 (5.00)	
Some difficulty	0 (0.00)	2 (10.00)	
Much difficulty	0 (0.00)	1 (5.00)	
Missing	2 (6.67)	2 (10.00)	
Washing dishes			0.1688
No difficulty	24 (80.00)	12 (60.00)	
A little difficulty	2 (6.67)	3 (15.00)	
Some difficulty	0 (0.00)	1 (5.00)	
Much difficulty	0 (0.00)	1 (5.00)	
Missing	4 (13.33)	3 (15.00)	

FACIT Dyspnea 10	CCP	Control	p-Value
Sweeping or mopping			0.0670
No difficulty	19 (63.33)	8 (40.00)	
A little difficulty	5 (16.67)	5 (25.00)	
Some difficulty	4 (13.33)	0 (0.00)	
Much difficulty	0 (0.00)	2 (10.00)	
Missing	2 (6.67)	5 (25.00)	
Making a bed			0.5572
No difficulty	20 (66.67)	10 (50.00)	
A little difficulty	6 (20.00)	4 (20.00)	
Some difficulty	2 (6.67)	2 (10.00)	
Much difficulty	0 (0.00)	1 (5.00)	
Missing	2 (6.67)	3 (15.00)	
Lifting something weighing 10-20 lbs			0.2545
No difficulty	17 (56.67)	7 (35.00)	
A little difficulty	7 (23.33)	4 (20.00)	
Some difficulty	3 (10.00)	5 (25.00)	
Much difficulty	1 (3.33)	2 (10.00)	
Missing	2 (6.67)	2 (10.00)	
Carrying something weighing 10-20 lbs from one room to another			0.4526
No difficulty	13 (43.33)	8 (40.00)	
A little difficulty	8 (26.67)	4 (20.00)	
Some difficulty	7 (23.33)	4 (20.00)	
Much difficulty	0 (0.00)	2 (10.00)	
Missing	2 (6.67)	2 (10.00)	
Walking for 1/2 a mile without stopping			0.7051
No difficulty	11 (36.67)	6 (30.00)	
A little difficulty	7 (23.33)	3 (15.00)	
Some difficulty	5 (16.67)	4 (20.00)	
Much difficulty	3 (10.00)	4 (20.00)	
Missing	4 (13.33)	3 (15.00)	

FACIT Dyspnea 10	CCP	Control	p-Value
**Raw functional limitation score**			0.1496
N	28	18	
Mean	4.65	8.30	
STD	5.66	9.37	
Min	0.00	0.00	
Q1	0.00	0.00	
Median	2.00	5.57	
Q3	6.33	12.00	
Max	18.00	29.00	
NMiss	2	2	

Table 10: FACIT Dyspnea 10 Item Short Form – Part 2 by transfused titer

FACIT Dyspnea 10	CCP	Control	p-Value
Number of patients in EFAS	14 (100.00)	16 (100.00)	
Dressing yourself without help			0.7833
No difficulty	10 (71.43)	13 (81.25)	
A little difficulty	2 (14.29)	2 (12.50)	
Some difficulty	1 (7.14)	0 (0.00)	
Missing	1 (7.14)	1 (6.25)	
Walking 50 steps on flat ground at a normal speed without stopping			0.3376
No difficulty	10 (71.43)	11 (68.75)	
A little difficulty	0 (0.00)	2 (12.50)	
Some difficulty	3 (21.43)	1 (6.25)	
Missing	1 (7.14)	2 (12.50)	
Walking 20 stairs without stopping			0.5166
No difficulty	5 (35.71)	9 (56.25)	
A little difficulty	5 (35.71)	3 (18.75)	
Some difficulty	2 (14.29)	3 (18.75)	
Much difficulty	1 (7.14)	0 (0.00)	
Missing	1 (7.14)	1 (6.25)	
Preparing meals			0.2063
No difficulty	11 (78.57)	15 (93.75)	
A little difficulty	2 (14.29)	0 (0.00)	
Missing	1 (7.14)	1 (6.25)	
Washing dishes			0.2031
No difficulty	10 (71.43)	14 (87.50)	
A little difficulty	2 (14.29)	0 (0.00)	
Missing	2 (14.29)	2 (12.50)	
Sweeping or mopping			0.3787
No difficulty	7 (50.00)	12 (75.00)	
A little difficulty	3 (21.43)	2 (12.50)	
Some difficulty	3 (21.43)	1 (6.25)	
Missing	1 (7.14)	1 (6.25)	
Making a bed			1.0000
No difficulty	9 (64.29)	11 (68.75)	
A little difficulty	3 (21.43)	3 (18.75)	
Some difficulty	1 (7.14)	1 (6.25)	
Missing	1 (7.14)	1 (6.25)	

FACIT Dyspnea 10	CCP	Control	p-Value
Lifting something weighing 10-20 lbs			0.1946
No difficulty	8 (57.14)	9 (56.25)	
A little difficulty	2 (14.29)	5 (31.25)	
Some difficulty	3 (21.43)	0 (0.00)	
Much difficulty	0 (0.00)	1 (6.25)	
Missing	1 (7.14)	1 (6.25)	
Carrying something weighing 10-20 lbs from one room to another			0.7081
No difficulty	5 (35.71)	8 (50.00)	
A little difficulty	4 (28.57)	4 (25.00)	
Some difficulty	4 (28.57)	3 (18.75)	
Missing	1 (7.14)	1 (6.25)	
Walking for 1/2 a mile without stopping			0.5257
No difficulty	4 (28.57)	7 (43.75)	
A little difficulty	5 (35.71)	2 (12.50)	
Some difficulty	2 (14.29)	3 (18.75)	
Much difficulty	1 (7.14)	2 (12.50)	
Missing	2 (14.29)	2 (12.50)	
Raw functional limitation score			0.4460
N	13	15	
Mean	5.55	3.87	
STD	6.62	4.78	
Min	0.00	0.00	
Q1	1.11	0.00	
Median	2.00	2.00	
Q3	6.00	6.67	
Max	18.00	15.00	
NMiss	1	1	

Table 11: FACIT Fatigue Scale, donors and patients

FACIT Fatigue Scale	Donors	Patient	p-Value
Number of patients	113 (100.00)	50 (100.00)	
I feel fatigued			0.2590
Not at all	45 (39.82)	15 (30.00)	
A little bit	36 (31.86)	17 (34.00)	
Somewhat	9 (7.96)	5 (10.00)	
Quite a bit	11 (9.73)	7 (14.00)	
Very much	1 (0.88)	3 (6.00)	
Missing	11 (9.73)	3 (6.00)	
I feel weak all over			0.0466
Not at all	61 (53.98)	20 (40.00)	
A little bit	27 (23.89)	13 (26.00)	
Somewhat	11 (9.73)	5 (10.00)	
Quite a bit	5 (4.42)	5 (10.00)	
Very much	0 (0.00)	3 (6.00)	
Missing	9 (7.96)	4 (8.00)	
I feel listless			0.0359
Not at all	53 (46.90)	24 (48.00)	
A little bit	34 (30.09)	7 (14.00)	
Somewhat	9 (7.96)	8 (16.00)	
Quite a bit	6 (5.31)	4 (8.00)	
Very much	2 (1.77)	4 (8.00)	
Missing	9 (7.96)	3 (6.00)	
I feel tired			0.0428
Not at all	33 (29.20)	18 (36.00)	
A little bit	41 (36.28)	13 (26.00)	
Somewhat	21 (18.58)	5 (10.00)	
Quite a bit	7 (6.19)	6 (12.00)	
Very much	2 (1.77)	5 (10.00)	
Missing	9 (7.96)	3 (6.00)	
I have trouble starting thing because I am tired			0.1306
Not at all	60 (53.10)	25 (50.00)	
A little bit	30 (26.55)	9 (18.00)	
Somewhat	10 (8.85)	4 (8.00)	
Quite a bit	4 (3.54)	6 (12.00)	
Very much	1 (0.88)	2 (4.00)	

FACIT Fatigue Scale	Donors	Patient	p-Value
Missing	8 (7.08)	4 (8.00)	
I have trouble finishing things because I am tired			0.0541
Not at all	65 (57.52)	22 (44.00)	
A little bit	24 (21.24)	13 (26.00)	
Somewhat	9 (7.96)	3 (6.00)	
Quite a bit	4 (3.54)	6 (12.00)	
Very much	1 (0.88)	3 (6.00)	
Missing	10 (8.85)	3 (6.00)	
I have energy			0.0025
Not at all	1 (0.88)	5 (10.00)	
A little bit	5 (4.42)	7 (14.00)	
Somewhat	24 (21.24)	13 (26.00)	
Quite a bit	57 (50.44)	14 (28.00)	
Very much	18 (15.93)	7 (14.00)	
Missing	8 (7.08)	4 (8.00)	
I am able to do my usual activities			<.0001
Not at all	1 (0.88)	8 (16.00)	
A little bit	2 (1.77)	7 (14.00)	
Somewhat	7 (6.19)	8 (16.00)	
Quite a bit	32 (28.32)	10 (20.00)	
Very much	63 (55.75)	13 (26.00)	
Missing	8 (7.08)	4 (8.00)	
I need sleep during the day			0.0824
Not at all	37 (32.74)	17 (34.00)	
A little bit	42 (37.17)	10 (20.00)	
Somewhat	15 (13.27)	7 (14.00)	
Quite a bit	8 (7.08)	6 (12.00)	
Very much	3 (2.65)	5 (10.00)	
Missing	8 (7.08)	5 (10.00)	
I am too tired to eat			0.0065
Not at all	99 (87.61)	35 (70.00)	
A little bit	3 (2.65)	4 (8.00)	
Somewhat	2 (1.77)	5 (10.00)	
Quite a bit	1 (0.88)	2 (4.00)	
Missing	8 (7.08)	4 (8.00)	

FACIT Fatigue Scale	Donors	Patient	p-Value
I need help doing my usual activities			0.0003
Not at all	95 (84.07)	30 (60.00)	
A little bit	7 (6.19)	4 (8.00)	
Somewhat	1 (0.88)	4 (8.00)	
Quite a bit	2 (1.77)	6 (12.00)	
Very much	0 (0.00)	2 (4.00)	
Missing	8 (7.08)	4 (8.00)	
I am frustrated by being too tired to do the things I want to do			0.0214
Not at all	74 (65.49)	25 (50.00)	
A little bit	19 (16.81)	7 (14.00)	
Somewhat	4 (3.54)	5 (10.00)	
Quite a bit	2 (1.77)	6 (12.00)	
Very much	6 (5.31)	3 (6.00)	
Missing	8 (7.08)	4 (8.00)	
I have to limit my social activity because I am tired			0.0066
Not at all	82 (72.57)	25 (50.00)	
A little bit	17 (15.04)	11 (22.00)	
Somewhat	1 (0.88)	0 (0.00)	
Quite a bit	5 (4.42)	7 (14.00)	
Very much	0 (0.00)	2 (4.00)	
Missing	8 (7.08)	5 (10.00)	
Fatigue scale score			0.0038
N	105	47	
Mean	43.65	37.17	
STD	8.46	13.63	
Min	16.00	3.00	
Q1	41.00	30.00	
Median	46.00	40.00	
Q3	50.00	49.00	
Max	52.00	52.00	
NMiss	8	3	

Table 12: FACIT Fatigue Scale by randomization group

FACIT Fatigue Scale	CCP	Control	p-Value
Number of patients in EFAS	30 (100.00)	20 (100.00)	
I feel fatigued			0.8935
Not at all	9 (30.00)	6 (30.00)	
A little bit	10 (33.33)	7 (35.00)	
Somewhat	3 (10.00)	2 (10.00)	
Quite a bit	5 (16.67)	2 (10.00)	
Very much	1 (3.33)	2 (10.00)	
Missing	2 (6.67)	1 (5.00)	
I feel weak all over			1.0000
Not at all	12 (40.00)	8 (40.00)	
A little bit	8 (26.67)	5 (25.00)	
Somewhat	3 (10.00)	2 (10.00)	
Quite a bit	3 (10.00)	2 (10.00)	
Very much	2 (6.67)	1 (5.00)	
Missing	2 (6.67)	2 (10.00)	
I feel listless			0.8839
Not at all	15 (50.00)	9 (45.00)	
A little bit	5 (16.67)	2 (10.00)	
Somewhat	4 (13.33)	4 (20.00)	
Quite a bit	2 (6.67)	2 (10.00)	
Very much	2 (6.67)	2 (10.00)	
Missing	2 (6.67)	1 (5.00)	
I feel tired			0.7344
Not at all	12 (40.00)	6 (30.00)	
A little bit	8 (26.67)	5 (25.00)	
Somewhat	2 (6.67)	3 (15.00)	
Quite a bit	4 (13.33)	2 (10.00)	
Very much	2 (6.67)	3 (15.00)	
Missing	2 (6.67)	1 (5.00)	
I have trouble starting thing because I am tired	15 (50.00)	10 (50.00)	0.6046
Not at all	6 (20.00)	3 (15.00)	
A little bit	3 (10.00)	1 (5.00)	
Somewhat	2 (6.67)	4 (20.00)	
Quite a bit	2 (6.67)	0 (0.00)	
Very much	2 (6.67)	2 (10.00)	
Missing			

FACIT Fatigue Scale	CCP	Control	p-Value
I have trouble finishing things because I am tired			0.7922
Not at all	14 (46.67)	8 (40.00)	
A little bit	8 (26.67)	5 (25.00)	
Somewhat	2 (6.67)	1 (5.00)	
Quite a bit	2 (6.67)	4 (20.00)	
Very much	2 (6.67)	1 (5.00)	
Missing	2 (6.67)	1 (5.00)	
I have energy			0.9070
Not at all	2 (6.67)	3 (15.00)	
A little bit	4 (13.33)	3 (15.00)	
Somewhat	8 (26.67)	5 (25.00)	
Quite a bit	8 (26.67)	6 (30.00)	
Very much	5 (16.67)	2 (10.00)	
Missing	3 (10.00)	1 (5.00)	
I am able to do my usual activities			0.2348
Not at all	4 (13.33)	4 (20.00)	
A little bit	4 (13.33)	3 (15.00)	
Somewhat	3 (10.00)	5 (25.00)	
Quite a bit	6 (20.00)	4 (20.00)	
Very much	11 (36.67)	2 (10.00)	
Missing	2 (6.67)	2 (10.00)	
I need sleep during the day			0.9574
Not at all	10 (33.33)	7 (35.00)	
A little bit	7 (23.33)	3 (15.00)	
Somewhat	4 (13.33)	3 (15.00)	
Quite a bit	3 (10.00)	3 (15.00)	
Very much	3 (10.00)	2 (10.00)	
Missing	3 (10.00)	2 (10.00)	
I am too tired to eat			0.0947
Not at all	24 (80.00)	11 (55.00)	
A little bit	1 (3.33)	3 (15.00)	
Somewhat	3 (10.00)	2 (10.00)	
Quite a bit	0 (0.00)	2 (10.00)	
Missing	2 (6.67)	2 (10.00)	

FACIT Fatigue Scale	CCP	Control	p-Value
**I need help doing my usual activities**			0.1883
Not at all	21 (70.00)	9 (45.00)	
A little bit	1 (3.33)	3 (15.00)	
Somewhat	1 (3.33)	3 (15.00)	
Quite a bit	4 (13.33)	2 (10.00)	
Very much	1 (3.33)	1 (5.00)	
Missing	2 (6.67)	2 (10.00)	
**I am frustrated by being too tired to do**			0.3712
**the things I want to do**	18 (60.00)	7 (35.00)	
Not at all	3 (10.00)	4 (20.00)	
A little bit	3 (10.00)	2 (10.00)	
Somewhat	2 (6.67)	4 (20.00)	
Quite a bit	2 (6.67)	1 (5.00)	
Very much	2 (6.67)	2 (10.00)	
Missing			
**I have to limit my social activity because**			0.5919
**I am tired**	17 (56.67)	8 (40.00)	
Not at all	5 (16.67)	6 (30.00)	
A little bit	4 (13.33)	3 (15.00)	
Quite a bit	1 (3.33)	1 (5.00)	
Very much	3 (10.00)	2 (10.00)	
Missing			
**Fatigue scale score**			0.3061
N	28	19	
Mean	38.87	34.68	
STD	13.05	14.43	
Min	8.00	3.00	
Q1	31.50	26.00	
Median	43.13	38.00	
Q3	49.00	47.00	
Max	52.00	51.00	
NMiss	2	1	

Table 13: FACIT Fatigue Scale by transfused titer

FACIT Fatigue Scale	Low titer Plasma	High titer Plasma	p-Value
Number of patients in EFAS	14 (100.00)	16 (100.00)	
I feel fatigued			0.8982
Not at all	4 (28.57)	5 (31.25)	
A little bit	4 (28.57)	6 (37.50)	
Somewhat	1 (7.14)	2 (12.50)	
Quite a bit	3 (21.43)	2 (12.50)	
Very much	1 (7.14)	0 (0.00)	
Missing	1 (7.14)	1 (6.25)	
I feel weak all over			0.6378
Not at all	5 (35.71)	7 (43.75)	
A little bit	3 (21.43)	5 (31.25)	
Somewhat	2 (14.29)	1 (6.25)	
Quite a bit	1 (7.14)	2 (12.50)	
Very much	2 (14.29)	0 (0.00)	
Missing	1 (7.14)	1 (6.25)	
I feel listless			0.6060
Not at all	6 (42.86)	9 (56.25)	
A little bit	3 (21.43)	2 (12.50)	
Somewhat	1 (7.14)	3 (18.75)	
Quite a bit	2 (14.29)	0 (0.00)	
Very much	1 (7.14)	1 (6.25)	
Missing	1 (7.14)	1 (6.25)	
I feel tired			0.2525
Not at all	4 (28.57)	8 (50.00)	
A little bit	6 (42.86)	2 (12.50)	
Somewhat	0 (0.00)	2 (12.50)	
Quite a bit	2 (14.29)	2 (12.50)	
Very much	1 (7.14)	1 (6.25)	
Missing	1 (7.14)	1 (6.25)	
I have trouble starting thing because I am tired			0.7582
Not at all	6 (42.86)	9 (56.25)	
A little bit	3 (21.43)	3 (18.75)	
Somewhat	1 (7.14)	2 (12.50)	
Quite a bit	1 (7.14)	1 (6.25)	
Very much	2 (14.29)	0 (0.00)	
Missing	1 (7.14)	1 (6.25)	

FACIT Fatigue Scale	Low titer Plasma	High titer Plasma	p-Value
I have trouble finishing things because I am tired			0.6334
Not at all	5 (35.71)	9 (56.25)	
A little bit	4 (28.57)	4 (25.00)	
Somewhat	1 (7.14)	1 (6.25)	
Quite a bit	1 (7.14)	1 (6.25)	
Very much	2 (14.29)	0 (0.00)	
Missing	1 (7.14)	1 (6.25)	
I have energy			0.8318
Not at all	1 (7.14)	1 (6.25)	
A little bit	1 (7.14)	3 (18.75)	
Somewhat	3 (21.43)	5 (31.25)	
Quite a bit	5 (35.71)	3 (18.75)	
Very much	2 (14.29)	3 (18.75)	
Missing	2 (14.29)	1 (6.25)	
I am able to do my usual activities			0.2163
Not at all	1 (7.14)	3 (18.75)	
A little bit	1 (7.14)	3 (18.75)	
Somewhat	3 (21.43)	0 (0.00)	
Quite a bit	4 (28.57)	2 (12.50)	
Very much	4 (28.57)	7 (43.75)	
Missing	1 (7.14)	1 (6.25)	
I need sleep during the day			0.0531
Not at all	2 (14.29)	8 (50.00)	
A little bit	5 (35.71)	2 (12.50)	
Somewhat	3 (21.43)	1 (6.25)	
Quite a bit	0 (0.00)	3 (18.75)	
Very much	2 (14.29)	1 (6.25)	
Missing	2 (14.29)	1 (6.25)	
I am too tired to eat			0.7833
Not at all	11 (78.57)	13 (81.25)	
A little bit	0 (0.00)	1 (6.25)	
Somewhat	2 (14.29)	1 (6.25)	
Missing	1 (7.14)	1 (6.25)	

FACIT Fatigue Scale	Low titer Plasma	High titer Plasma	p-Value
I need help doing my usual activities			0.3362
Not at all	9 (64.29)	12 (75.00)	
A little bit	1 (7.14)	0 (0.00)	
Somewhat	0 (0.00)	1 (6.25)	
Quite a bit	3 (21.43)	1 (6.25)	
Very much	0 (0.00)	1 (6.25)	
Missing	1 (7.14)	1 (6.25)	
I am frustrated by being too tired to do the things I want to do			0.5876
Not at all	7 (50.00)	11 (68.75)	
A little bit	2 (14.29)	1 (6.25)	
Somewhat	1 (7.14)	2 (12.50)	
Quite a bit	2 (14.29)	0 (0.00)	
Very much	1 (7.14)	1 (6.25)	
Missing	1 (7.14)	1 (6.25)	
I have to limit my social activity because I am tired			1.0000
Not at all	8 (57.14)	9 (56.25)	
A little bit	2 (14.29)	3 (18.75)	
Quite a bit	2 (14.29)	2 (12.50)	
Very much	1 (7.14)	0 (0.00)	
Missing	1 (7.14)	2 (12.50)	
Fatigue scale score			0.4916
N	13	15	
Mean	37.00	40.48	
STD	15.61	10.66	
Min	8.00	16.00	
Q1	30.00	33.00	
Median	42.00	44.00	
Q3	49.00	49.00	
Max	52.00	52.00	
NMiss	1	1	

Table 14: Laboratory parameters CCP donors and trial patients

Lab parameter	Donors	Patients	p-value
**D-Dimers (mg/l)**	0.27 (0.19-0.37(	0.43 (0.25-0.85)	0.02
**Fibrinogen (mg/dl)**	290 (240-350)	330 (295-400)	0.03
**CRP (mg/l)**	1.8 (0.8-4.0)	5.6 (1.5-9.3)	0.002
**Ferritin (µg/l)**	62 (37-126)	120 (82-213)	0.03
**IL-6 (pg/ml)**	1.6 (1.4-2.2)	3.1 (1.8-4.7(	0.004
**Pro-NT BNP (pg/ml)**	45 (29-67)	95 (29-223)	0.03

Table 15: Classification to assess disease severity of CCP donors.

Severity	Symptoms
asymptomatic	No symptoms
mild	The virus affects mainly the upper respiratory tract. Key symptoms are temperature with or without cough
moderate	Inflammation lower down in the lungs. Key symptoms are breathless, increased heart rate, temperature and cough
severe	At least one of the following:<br>- Resporatory rate ≥ 30 breaths / min under ambient air<br>- Requirement of any type of ventilation suppport<br>- Needs ICU treatment

Methods: Propensity score matching

To better control the donor and patient population a subset was created with a 1:1 matching of patients and donors according to the following baseline criteria:

- age at extended follow up visit (continuous variable)
- BMI (categories <30 kg/m^2, 30-<35 kg/m^2, 35-<40 kg/m^2, ≥40 kg/m^2)
- sex (male; female)

Propensity score matching was performed by using the SAS procedure PROC PSMATCH with default settings using greedy nearest neighbor matching to identify matched patients.

Table 16: Propensity Score matching: Donor / Patients

Demographics	Donor	Patient	p-Value
**Number**	26 (100.00)	26 (100.00)	
**Gender**			1.0000
**Male**	22 (84.62)	21 (80.77)	
**Female**	4 (15.38)	5 (19.23)	
**Age [years]**			0.2856
**N**	26	26	
**Min**	31.00	30.00	
**Q1**	46.00	47.00	
**Median**	51.50	54.00	
**Q3**	55.00	56.00	
**Max**	61.00	70.00	
**NMiss**	0	0	
**BMI (kg/m^2)**			0.6247
**N**	22	23	
**Min**	21.31	20.60	
**Q1**	25.69	26.56	
**Median**	29.46	29.74	
**Q3**	31.15	33.90	
**Max**	38.82	41.21	
**NMiss**	4	3	

Table 17: Medical events and symptoms in propensity score matched cohort

Medical Events/Symptoms	Donor	Patient	p-Value	Total
Number of patients	26 (100.00)	26 (100.00)		52 (100.00)
Abdominal pain			0.3497	
Without Event	25 (96.15)	22 (84.62)		47 (90.38)
With Event	1 (3.85)	4 (15.38)		5 (9.62)
Grade 1	1 (3.85)	3 (11.54)		4 (7.69)
Grade 2	0 (0.00)	1 (3.85)		1 (1.92)
Allergic rhinitis				
Without Event	26 (100.00)	26 (100.00)		52 (100.00)
Alopecia			1.0000	
Without Event	23 (88.46)	23 (88.46)		
With Event	3 (11.54)	3 (11.54)		
Grade 1	1 (3.85)	2 (7.69)		
Grade 2	2 (7.69)	1 (3.85)		
Amnesia			0.4902	
Without Event	26 (100.00)	24 (92.31)		50 (96.15)
With Event	0 (0.00)	2 (7.69)		2 (3.85)
Grade 1	0 (0.00)	2 (7.69)		2 (3.85)
Anorexia			0.2353	
Without Event	26 (100.00)	23 (88.46)		49 (94.23)
With Event	0 (0.00)	3 (11.54)		3 (5.77)
Grade 1	0 (0.00)	3 (11.54)		3 (5.77)
Anosmia			0.2203	
Without Event	16 (61.54)	21 (80.77)		37 (71.15)
With Event	10 (38.46)	5 (19.23)		15 (28.85)
Grade 1	10 (38.46)	5 (19.23)		15 (28.85)
Arthralgia			0.4902	
Without Event	26 (100.00)	24 (92.31)		50 (96.15)
With Event	0 (0.00)	2 (7.69)		2 (3.85)
Grade 1	0 (0.00)	2 (7.69)		2 (3.85)
Atrial fibrillation			1.0000	
Without Event	26 (100.00)	25 (96.15)		51 (98.08)
With Event	0 (0.00)	1 (3.85)		1 (1.92)
Grade 1	0 (0.00)	1 (3.85)		1 (1.92)

Medical Events/Symptoms	Donor	Patient	p-Value	Total
**Back pain**			1.0000	
Without Event	25 (96.15)	25 (96.15)		50 (96.15)
With Event	1 (3.85)	1 (3.85)		2 (3.85)
Grade 1	0 (0.00)	1 (3.85)		1 (1.92)
Grade 2	1 (3.85)	0 (0.00)		1 (1.92)
**Bone pain**			0.1104	
Without Event	22 (84.62)	26 (100.00)		48 (92.31)
With Event	4 (15.38)	0 (0.00)		4 (7.69)
Grade 1	3 (11.54)	0 (0.00)		3 (5.77)
Grade 2	1 (3.85)	0 (0.00)		1 (1.92)
**Bronchial infection**				
Without Event	26 (100.00)	26 (100.00)		52 (100.00)
**Chest pain - cardiac**				
Without Event	26 (100.00)	26 (100.00)		52 (100.00)
**Concentration impairment**				
Without Event	26 (100.00)	26 (100.00)		52 (100.00)
**Confusion**			0.2353	
Without Event	26 (100.00)	23 (88.46)		49 (94.23)
With Event	0 (0.00)	3 (11.54)		3 (5.77)
Grade 1	0 (0.00)	1 (3.85)		1 (1.92)
Grade 2	0 (0.00)	2 (7.69)		2 (3.85)
**Conjunctivitis**			0.2353	
Without Event	26 (100.00)	23 (88.46)		49 (94.23)
With Event	0 (0.00)	3 (11.54)		3 (5.77)
Grade 1	0 (0.00)	2 (7.69)		2 (3.85)
Grade 3	0 (0.00)	1 (3.85)		1 (1.92)
**Constipation**				
Without Event	26 (100.00)	26 (100.00)		52 (100.00)
**Cough**			0.7645	
Without Event	17 (65.38)	19 (73.08)		36 (69.23)
With Event	9 (34.62)	7 (26.92)		16 (30.77)
Grade 1	7 (26.92)	6 (23.08)		13 (25.00)
Grade 2	2 (7.69)	1 (3.85)		3 (5.77)
**Depression**				52 (100.00)
Without Event	26 (100.00)	26 (100.00)		

Medical Events/Symptoms	Donor	Patient	p-Value	Total
Diarrhea			0.0993	
Without Event	25 (96.15)	20 (76.92)		45 (86.54)
With Event	1 (3.85)	6 (23.08)		7 (13.46)
Grade 1	1 (3.85)	6 (23.08)		7 (13.46)
Dizziness			0.2485	
Without Event	24 (92.31)	20 (76.92)		44 (84.62)
With Event	2 (7.69)	6 (23.08)		8 (15.38)
Grade 1	2 (7.69)	6 (23.08)		8 (15.38)
Dry eye			0.3497	
Without Event	25 (96.15)	22 (84.62)		47 (90.38)
With Event	1 (3.85)	4 (15.38)		5 (9.62)
Grade 1	1 (3.85)	3 (11.54)		4 (7.69)
Grade 2	0 (0.00)	1 (3.85)		1 (1.92)
Dysesthesia				
Without Event	26 (100.00)	26 (100.00)		52 (100.00)
Dysgeusia			0.3487	
Without Event	17 (65.38)	21 (80.77)		38 (73.08)
With Event	9 (34.62)	5 (19.23)		14 (26.92)
Grade 1	8 (30.77)	4 (15.38)		12 (23.08)
Grade 2	1 (3.85)	1 (3.85)		2 (3.85)
Dyspnea			0.4043	
Without Event	16 (61.54)	12 (46.15)		28 (53.85)
With Event	10 (38.46)	14 (53.85)		24 (46.15)
Grade 1	7 (26.92)	7 (26.92)		14 (26.92)
Grade 2	2 (7.69)	6 (23.08)		8 (15.38)
Grade 3	1 (3.85)	1 (3.85)		2 (3.85)
Eczema			1.0000	
Without Event	25 (96.15)	25 (96.15)		50 (96.15)
With Event	1 (3.85)	1 (3.85)		2 (3.85)
Grade 1	1 (3.85)	0 (0.00)		1 (1.92)
Grade 2	0 (0.00)	1 (3.85)		1 (1.92)
Eye infection			1.0000	
Without Event	25 (96.15)	26 (100.00)		51 (98.08)
With Event	1 (3.85)	0 (0.00)		1 (1.92)
Grade 2	1 (3.85)	0 (0.00)		1 (1.92)

Medical Events/Symptoms	Donor	Patient	p-Value	Total
**Fatigue**			1.0000	
Without Event	16 (61.54)	17 (65.38)		33 (63.46)
With Event	10 (38.46)	9 (34.62)		19 (36.54)
Grade 1	5 (19.23)	5 (19.23)		10 (19.23)
Grade 2	3 (11.54)	3 (11.54)		6 (11.54)
Grade 3	2 (7.69)	1 (3.85)		3 (5.77)
**Fever**			0.2485	
Without Event	20 (76.92)	24 (92.31)		44 (84.62)
With Event	6 (23.08)	2 (7.69)		8 (15.38)
Grade 1	3 (11.54)	1 (3.85)		4 (7.69)
Grade 2	3 (11.54)	0 (0.00)		3 (5.77)
Grade 3	0 (0.00)	1 (3.85)		1 (1.92)
**Fracture**			1.0000	
Without Event	25 (96.15)	26 (100.00)		51 (98.08)
With Event	1 (3.85)	0 (0.00)		1 (1.92)
Grade 2	1 (3.85)	0 (0.00)		1 (1.92)
**Generalized muscle weakness**				
Without Event	26 (100.00)	26 (100.00)		52 (100.00)
**Headache**			0.4986	
Without Event	19 (73.08)	22 (84.62)		41 (78.85)
With Event	7 (26.92)	4 (15.38)		11 (21.15)
Grade 1	5 (19.23)	2 (7.69)		7 (13.46)
Grade 2	0 (0.00)	2 (7.69)		2 (3.85)
Grade 3	2 (7.69)	0 (0.00)		2 (3.85)
**Hypersomnia**			1.0000	
Without Event	20 (76.92)	20 (76.92)		40 (76.92)
With Event	6 (23.08)	6 (23.08)		12 (23.08)
Grade 1	3 (11.54)	2 (7.69)		5 (9.62)
Grade 2	3 (11.54)	3 (11.54)		6 (11.54)
Grade 3	0 (0.00)	1 (3.85)		1 (1.92)
**Hypertension**				
Without Event	26 (100.00)	26 (100.00)		52 (100.00)
**Immune system disorders - Other, specify**				
Without Event	26 (100.00)	26 (100.00)		52 (100.00)

Medical Events/Symptoms	Donor	Patient	p-Value	Total
Infections and infestations - Other, specify				
Without Event	26 (100.00)	26 (100.00)		52 (100.00)
Insomnia			1.0000	
Without Event	26 (100.00)	25 (96.15)		51 (98.08)
With Event	0 (0.00)	1 (3.85)		1 (1.92)
Grade 1	0 (0.00)	1 (3.85)		1 (1.92)
Investigations - Other, specify				
Without Event	26 (100.00)	26 (100.00)		52 (100.00)
Memory impairment			0.5653	
Without Event	18 (69.23)	15 (57.69)		33 (63.46)
With Event	8 (30.77)	11 (42.31)		19 (36.54)
Grade 1	7 (26.92)	7 (26.92)		14 (26.92)
Grade 2	1 (3.85)	3 (11.54)		4 (7.69)
Grade 3	0 (0.00)	1 (3.85)		1 (1.92)
Meningismus				
Without Event	26 (100.00)	26 (100.00)		52 (100.00)
Muscle cramp			1.0000	
Without Event	25 (96.15)	26 (100.00)		51 (98.08)
With Event	1 (3.85)	0 (0.00)		1 (1.92)
Grade 2	1 (3.85)	0 (0.00)		1 (1.92)
Myalgia			1.0000	
Without Event	24 (92.31)	25 (96.15)		49 (94.23)
With Event	2 (7.69)	1 (3.85)		3 (5.77)
Grade 1	1 (3.85)	1 (3.85)		2 (3.85)
Grade 2	1 (3.85)	0 (0.00)		1 (1.92)
Myocardial infarction				
Without Event	26 (100.00)	26 (100.00)		52 (100.00)
Nausea			0.1104	
Without Event	26 (100.00)	22 (84.62)		48 (92.31)
With Event	0 (0.00)	4 (15.38)		4 (7.69)
Grade 1	0 (0.00)	3 (11.54)		3 (5.77)
Grade 2	0 (0.00)	1 (3.85)		1 (1.92)

Medical Events/Symptoms	Donor	Patient	p-Value	Total
**Nervous system disorders - Other, specify**				
Without Event	26 (100.00)	26 (100.00)		52 (100.00)
**Non-cardiac chest pain**			1.0000	
Without Event	22 (84.62)	21 (80.77)		43 (82.69)
With Event	4 (15.38)	5 (19.23)		9 (17.31)
Grade 1	3 (11.54)	1 (3.85)		4 (7.69)
Grade 2	1 (3.85)	3 (11.54)		4 (7.69)
Grade 3	0 (0.00)	1 (3.85)		1 (1.92)
**Pain**			0.1906	
Without Event	25 (96.15)	21 (80.77)		46 (88.46)
With Event	1 (3.85)	5 (19.23)		6 (11.54)
Grade 1	0 (0.00)	3 (11.54)		3 (5.77)
Grade 2	1 (3.85)	2 (7.69)		3 (5.77)
**Pain in extremity**			0.1104	
Without Event	26 (100.00)	22 (84.62)		48 (92.31)
With Event	0 (0.00)	4 (15.38)		4 (7.69)
Grade 1	0 (0.00)	3 (11.54)		3 (5.77)
Grade 3	0 (0.00)	1 (3.85)		1 (1.92)
**Palpitations**			0.7030	
Without Event	23 (88.46)	21 (80.77)		44 (84.62)
With Event	3 (11.54)	5 (19.23)		8 (15.38)
Grade 1	3 (11.54)	4 (15.38)		7 (13.46)
Grade 2	0 (0.00)	1 (3.85)		1 (1.92)
**Productive cough**			1.0000	
Without Event	25 (96.15)	24 (92.31)		49 (94.23)
With Event	1 (3.85)	2 (7.69)		3 (5.77)
Grade 1	1 (3.85)	2 (7.69)		3 (5.77)
**Rash maculo-papular**			0.4902	50 (96.15)
Without Event	26 (100.00)	24 (92.31)		2 (3.85)
With Event	0 (0.00)	2 (7.69)		1 (1.92)
Grade 1	0 (0.00)	1 (3.85)		1 (1.92)
Grade 2	0 (0.00)	1 (3.85)		

Medical Events/Symptoms	Donor	Patient	p-Value	Total
Restlessness				
Without Event	26 (100.00)	26 (100.00)		52 (100.00)
Rhinitis infective			1.0000	
Without Event	24 (92.31)	24 (92.31)		48 (92.31)
With Event	2 (7.69)	2 (7.69)		4 (7.69)
Grade 2	2 (7.69)	2 (7.69)		4 (7.69)
Sinus tachycardia				
Without Event	26 (100.00)	26 (100.00)		52 (100.00)
Sleep apnea			1.0000	
Without Event	26 (100.00)	25 (96.15)		51 (98.08)
With Event	0 (0.00)	1 (3.85)		1 (1.92)
Grade 2	0 (0.00)	1 (3.85)		1 (1.92)
Surgical and medical procedures - Other, specify				
Without Event	26 (100.00)	26 (100.00)		52 (100.00)
Tinnitus				
Without Event	26 (100.00)	26 (100.00)		52 (100.00)
Upper respiratory infection				
Without Event	26 (100.00)	26 (100.00)		52 (100.00)
Vaginal infection				52 (100.00)
Without Event	26 (100.00)	26 (100.00)		
Ventricular arrhythmia			0.6098	
Without Event	25 (96.15)	23 (88.46)		48 (92.31)
With Event	1 (3.85)	3 (11.54)		4 (7.69)
Grade 1	1 (3.85)	2 (7.69)		3 (5.77)
Grade 2	0 (0.00)	1 (3.85)		1 (1.92)
Vision decreased				
Without Event	26 (100.00)	26 (100.00)		52 (100.00)
Vomiting			1.0000	
Without Event	26 (100.00)	25 (96.15)		51 (98.08)
With Event	0 (0.00)	1 (3.85)		1 (1.92)
Grade 1	0 (0.00)	1 (3.85)		1 (1.92)

Medical Events/Symptoms	Donor	Patient	p-Value	Total
**Weight loss**			0.0098	
**Without Event**	26 (100.00)	19 (73.08)		45 (86.54)
**With Event**	0 (0.00)	7 (26.92)		7 (13.46)
**Grade 1**	0 (0.00)	5 (19.23)		5 (9.62)
**Grade 2**	0 (0.00)	2 (7.69)		2 (3.85)

Supplemental Figure 3: Post-COVID-19 Scale and change in socioeconomic status in the propensity score matched cohort

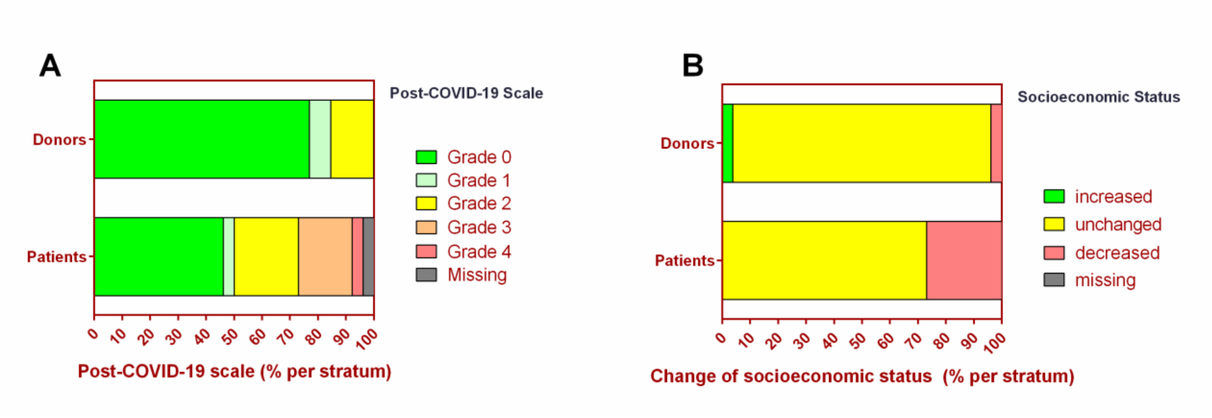

Supplemental Figure 3: Post-COVID-19 Scale (panel A) and change in socioeconomic status (panel B) in the propensity score matched cohort.

The bar section show the relative proportion of individuals with the respective grade or status.
(A) Donors showed less impairment than patients did in the Post-COVID-19 scale (p=0.038).
(B) Donors remained in better socioeconomic status that patients (p=0.0496).

For the demographic characteristics of the propensity score matched patient and donor cohort see Table 16.

Supplemental Figure 4: QoL Scores of the propensity score matched cohort

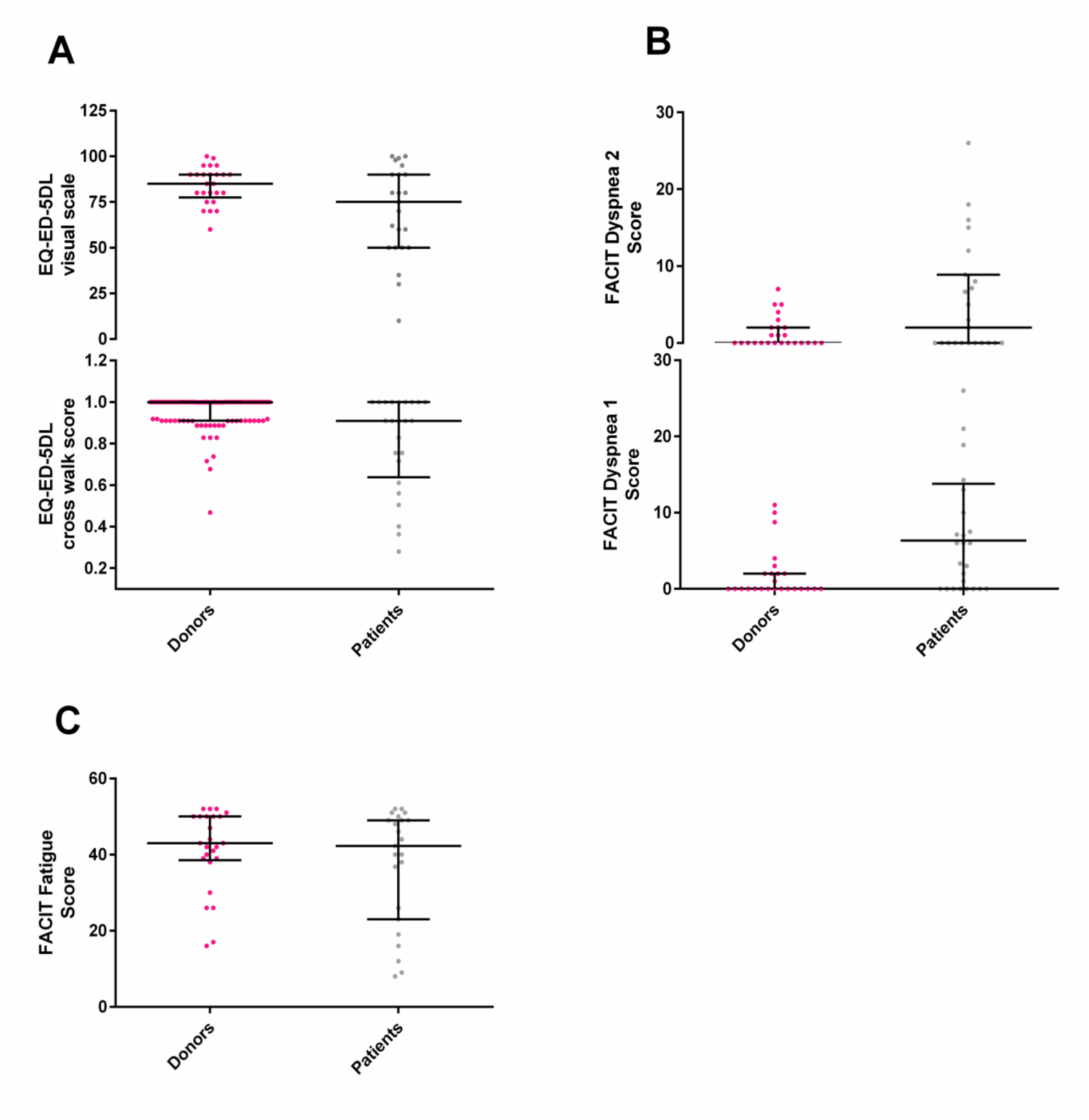

Supplemental Figure 4: QoL Scores of the propensity score matched cohort (patients, n=26 and donors, n=26)

EQ-5D-5L visual scale and cross walk score (panel A), FACIT Dyspnea 1 score 1and Dyspnea 2 score (panel B) and FACIT Fatigue Score (panel C).

(A) Donors were well than patients in the EQ-ED-5DL visual scale (p=0.0264) and EQ-ED-5DL cross walk score (p=0.0264).

(B) FACIT Dyspnea scores were better in donors than patients (Dyspnea score 1 (p=0.0570) and Dyspnea score 2 (p=0.0132)).

(C) The FACIT Fatigue Scores were not significantly difference between the groups (p=0.2700).

For the demographic characteristics of the propensity score matched patient and donor cohort see Table 16.
